# Estrogen Receptor Alpha Signaling Is Responsible for the Female Sex Bias in the Loss of Tolerance and Immune Cell Activation Induced by the Lupus Susceptibility Locus *Sle1b*

**DOI:** 10.3389/fimmu.2020.582214

**Published:** 2020-11-10

**Authors:** Jared H. Graham, Shayla D. Yoachim, Karen A. Gould

**Affiliations:** Department of Genetics, Cell Biology & Anatomy, University of Nebraska Medical Center, Omaha, NE, United States

**Keywords:** estrogen receptor, female, sex bias, lupus, immune tolerance, B cell activation, T cell activation

## Abstract

The dramatic female sex bias observed in human lupus is thought to be due, at least in part, to estrogens. Using mouse models, we have shown that estrogens, acting through estrogen receptor alpha (ERα) promote lupus development and contribute significantly to the female sex bias observed in this disease. C57Bl/6 (B6) mice carrying the lupus susceptibility locus *Sle1* locus exhibit immune cell hyperactivation and loss of tolerance, and the action of *Sle1* displays a strong female sex bias. Previously, we showed that disruption of ERα completely eliminates the female sex bias in the effects of *Sle1*. Here we report that ERα signaling selectively modulates the action of *Sle1b*, one of the three subloci that together constitute *Sle1*. We observed that disruption of ERα signaling attenuated T cell hyperactivation, formation of spontaneous germinal centers, loss of tolerance, and the development of anti-chromatin autoantibodies in B6.*Sle1b* female mice, but had no impact on these phenotypes in B6.*Sle1b* male mice. In fact, disruption of ERα completely abolished the female sex bias that is seen in each of these phenotypes in B6.*Sle1b* mice. Strikingly, *Sle1b*-induced B cell hyperactivation, a female sex-specific manifestation of *Sle1b*, was completely abrogated by disruption of ERα in B6.*Sle1b* females. Altogether, these results demonstrate that ERα signaling is responsible for the female sex bias in the actions of *Sle1b*, and is absolutely required for the female-specific B cell hyperactivation phenotype associated with this lupus susceptibility locus. By contrast, we found that ERα signaling had no impact on *Sle1a*, the other *Sle1* sublocus that exerts effects that show a female sex bias.

## Introduction

Approximately 90% of lupus patients are women, and this striking female sex bias is thought to be due, in part, to the action of endogenous estrogens. Although there is abundant evidence from epidemiological studies linking enhanced lupus risk and increased lupus symptoms with exposure to endogenous and exogenous estrogens (1–9) the cellular and molecular basis for these effects are not understood.

Much of our understanding of how estrogens may promote lupus has come from studies in lupus prone mice, such as (NZB × NZW)F1 mice, in which lupus development shows a strong female sex bias (10, 11). We showed previously that disruption of estrogen receptor α (ERα) eliminated the female sex bias in the development of lupus nephritis in (NZB × NZW)F1 mice and dramatically attenuated the loss of tolerance to nuclear antigens and the development of autoantibodies (12). These data indicate that the female sex bias in this model of lupus is largely the result of estrogens, acting *via* ERα. This initial study was conducted using a global knockout of ERα, but in a subsequent study using a B cell-specific deletion of ERα, we showed that ERα acts, at least in part, in a B cell intrinsic manner to control B cell activation, autoantibody development, and the development of lupus nephritis (13).

Loss of tolerance to chromatin is thought to represent an initial step in lupus pathogenesis (14, 15). In (NZB × NZW)F1 mice, the NZW-derived lupus susceptibility allele of the *Sle1* locus is one of the alleles that drives this initial loss of tolerance (16–18). B6.*Sle1* congenic mice, in which the NZW-derived *Sle1* allele is carried on the non-autoimmune C57BL/6 (B6) genetic background, lose tolerance to chromatin, develop anti-chromatin IgG autoantibodies, and display B and T cell hyperactivation (19–21). Both the loss of tolerance and immune cell hyperactivation phenotypes in B6.*Sle1* mice show a strong female sex bias; Compared to their male counterparts, a greater proportion of B6.*Sle1* females lose tolerance and develop anti-chromatin IgG autoantibodies, and a greater proportion of B cells and T cells in B6.*Sle1* females express activation markers and/or express higher levels of activation markers (22–24). In fact, we have shown that B cell activation is a female-specific manifestation of *Sle1* (24). Furthermore, we found that the female sex bias in *Sle1*-induced loss of tolerance to chromatin and immune cell hyperactivation is eliminated by disruption of *ERα* (24). Likewise, the B cell hyperactivation phenotype in B6.*Sle1* females is abrogated by disruption of ERα, indicating that this female sex specific phenotype associated with *Sle1* is dependent upon *ERα* (24). Ovariectomy, which removes the primary source of estrogens in females, also eliminates the female sex bias in the effects of *Sle1* suggesting that estrogen-dependent actions of ERα are responsible for the female sex bias in *Sle1* (24).

The *Sle1* locus contains at least three distinct subloci, *Sle1a*, *Sle1b*, and *Sle1c* (23, 25). Although *Sle1a*, *Sle1b*, and *Sle1c* can each independently induce loss of tolerance to chromatin, the magnitude of these effects and the underlying cellular mechanisms are distinct for each sublocus (23, 25–29). The *Sle1a* locus is associated with the development of activated, autoreactive CD4^+^ T cells, and although *Sle1a-*induced loss of tolerance to chromatin was described as being more pronounced in female mice than in male mice, this difference fell short of statistical significance (23, 26–29). By contrast, the actions of *Sle1b* show a robust female sex bias (23, 30, 31). The *Sle1b* locus is associated with loss of tolerance, B and T cell hyperactivation, and alterations in the germinal center checkpoint (23, 30, 31). The *Sle1c* locus is also associated with B and T cell activation, but there is little evidence that the action of *Sle1c* shows a female sex bias (23, 26–29).

Based on our finding that ERα is required for the female sex bias in *Sle1*-induced loss of tolerance and immune cell hyperactivation and the observation that the effects of the *Sle1* subloci show varying degrees of female sex bias, we hypothesize that ERα signaling synergizes with the pathways controlled by certain *Sle1* subloci to preferentially enhance loss of tolerance, immune cell activation, and ultimately the development of lupus in females. To test this hypothesis, we examined the impact of targeted disruption of *ERα* on the phenotype in B6.*Sle1a* and B6.*Sle1b* congenic mice. Although the actions of *Sle1a* do show some degree of female sex bias, this sex bias was not impacted by disruption of ERα. By contrast, the female sex bias in the effects of *Sle1b* were completely eliminated by disruption of ERα, suggesting that ERα signaling, selectively impacts the pathways controlled by *Sle1b* and potentiates the actions of the lupus susceptibility locus *Sle1b* in females.

## Materials and Methods

### Care and Treatment of Mice

The ERα knockout strain (B6.129-*Esr1^tm1Ks^* ^k^ or B6.*ERα*) (32) was originally obtained from Dennis Lubahn. The B6.*Sle1a* and B6.*Sle1b* congenic strains (23, 24) were provided by Laurence Morel. Animals were housed under controlled temperature, humidity, and 14 h light/10 h dark lighting conditions in a facility accredited by the American Association for Accreditation of Laboratory Animal Care and operated in accordance with the standards outlined in Guide for the Care and Use of Laboratory Animals (The National Academies Press, 1996). Mice were provided Harlan irradiated rodent diet 7904 (Harlan Teklad, Madison, WI), which contains soy, milk, and meat-based protein sources, and allowed to feed ad libitum.

B6 females heterozygous for targeted disruption of the *ERα* gene (*ERα^+/-^*) were crossed to B6.*Sle1a* or B6.*Sle1b* congenic males. The resulting *ERα^+/-^* males were backcrossed to B6.*Sle1a* or B6.*Sle1b* females respectively. Resulting *ERα^+/-^* offspring were genotyped at markers that are polymorphic between the NZW and B6 strains and flank either the *Sle1a* or *Sle1b* congenic interval to identify mice that were homozygous for NZW alleles throughout each interval. For the *Sle1a* locus, the markers *D1Mit15* and *D1Mit353* were used whereas for the *Sle1b* locus, the markers *D1Mit113* and *D1Mit206* were used (23). The selected B6.*Sle1a*.*ERα^+/-^* mice were interbred to generate the experimental mice for the studies involving *Sle1a*. Likewise, the selected B6.*Sle1b*.*ERα^+/-^* mice were interbred to generate the experimental mice for the studies involving *Sle1b*. PCR-based assays to determine genotype at *ERα* as well as at markers on chromosome 1 were performed as described previously (12, 13, 24).

### Serological Analysis

Autoantibodies were quantified by ELISA using serum isolated from blood collected at ~5 months of age from experimental mice *via* the saphenous vein and stored at -80C. The serum anti-chromatin IgG autoantibody concentrations were determined using plates prepared as described previously (12, 24, 33). Autoantibody levels in these samples were quantitated in arbitrary ELISA units (U/µl) based on a standard curve generated by serial dilution of a positive control sample that was made by pooling serum from a group of (NZB x NZW)F1 females with lupus nephritis. The threshold for a positive autoantibody measurement in the experimental mice was fixed at two standard deviations above the mean of a group of age-matched, control B6 female mice (12, 23, 24, 33). Total serum concentrations of antibodies of each isotype were determined using the clonotyping kit (Southern Biotech, Birmingham, AL) according to the manufacturer’s instructions. Serial dilutions of serum samples ranging from 1:100 to 1:2000 were used for measurement of autoantibody concentrations. Serial dilution up to 1:50,000 were used for measurement of total serum immunoglobulins. Serum estradiol and testosterone concentrations were determined by ELISA according to the manufacturer’s instructions (Alpha Diagnostics International, San Antonio, TX). Samples were assayed in duplicate or triplicate. All optical density measurements were made using a BioRad 680 Microplate reader and Microplate Manager software, version 5.2.1 (Hercules, CA).

### Flow Cytometry

The antibodies used for flow cytometry included: CD4-PE (RM4-5), CD4-v450 (RM4-5), CD5-PE (53-7.3), CD8-APC (53-6.7), CD19-FITC (ID3), CD22-PE (Cy34.1), CD45R/B220-APC (RA3-6B2), CD62L-APC (MEL-14), CD69-FITC(H1.2F3), CD86-PE (GL1), CD93(AA4.1), CD95-PE.Cy7 (Jo2), CD134-Biotin (OX-86), CXCR5-PE.Cy7 (2G8), PD-1-APC (J43), and PNA-FITC (L7281) (all from BD Biosciences, San Jose, CA), IgM-FITC) (R6-60.2 from Southern Biotech), IgD-APC-Cy7 (11-26c.2a from BioLegend, San Diego, CA, USA), CD21/CD35-eFlour450 [eBio4F3 (4E3)], and CD23-PE-Cy7 (B3B4) (both from eBioscience Inc., San Diego, CA, USA). Biotinylated antibodies were detected using FITC-conjugated streptavidin (BD Biosciences). Flow cytometric analysis was performed using various combinations of these antibodies on single cell suspensions of splenocytes. Stained cells were analyzed in the UNMC Flow Cytometry Research Facility using the BD LSR II flow cytometer. Data were analyzed using FACSDiva software, version 8.0.2 (BD Biosciences). For flow cytometry analyses, splenocytes were collected from mice that were 5–6 months of age.

### Immunohistochemistry

A segment of each spleen collected at sacrifice from mice 5–6 months of age was fixed in 10% formalin, processed, embedded in paraffin, and sectioned. After deparaffinization and rehydration, slides were incubated in 0.3% H_2_O_2_ for 30 min, washed and incubated for 20 min in 95°C citrate buffer (Vector Laboratories, Burlingame, CA). Slides were blocked for 20 min with Carbo-free blocking solution (Vector Labs), and incubated with a biotinylated anti-PNA antibody (Vector Labs) diluted in phosphate-buffered saline for 30 min. Antibody binding was visualized using Vectastain Elite ABC and DAB reagents (Vector Labs). The number and size of germinal centers based upon PNA staining were quantified using Zeiss Zen Pro software (v4.6.3.0; Carl Zeiss, Thornwood, NY). Quantification was performed by a single observer (J.H.G.) who was blinded to the sex and genotype of the sample.

### Statistical Analysis

Comparisons were performed using Fishers exact test (for comparisons of proportions of mice displaying loss of tolerance), independent samples t-test (for comparison of anti-chromatin IgG concentrations in female and male B6.*Sle1a* mice), or one-way ANOVA with Tukey’s *post hoc* test (for all other comparisons). Statistical analyses were performed using SPSS software (version 26.0). A two-sided P ≤ 0.05 was considered significant, and two-sided p-values are provided. 

## Results

### T Cell Hyperactivation but Not Loss of Tolerance to Chromatin Shows a Female Sex Bias in B6*.Sle1a* Mice

**Figure 1:**
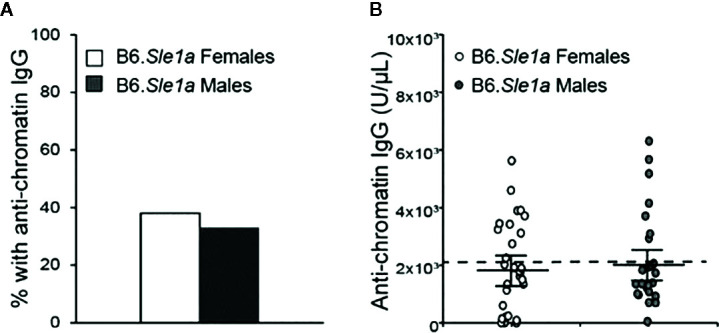
Loss of tolerance in B6. *Sle1a* congenic mice does not show a significant female sex bias. **(A)** The proportion of B6.*Sle1a* female and male mice producing anti-chromatin IgG autoantibodies at 5 months of age is shown. **(B)** The concentration of anti-chromatin IgG autoantibodies in the B6.*Sle1a* congenic female (N=29) and male (N=24) mice evaluated is shown. The dashed line represents the threshold used to designate a positive autoantibody titer in the experimental mice. This threshold was set at 2 standard deviations above the mean of a group of age-matched control B6 mice as has been described previously (23, 24). The longer black horizontal bar indicates the mean for each group, and the shorter black bars indicate the standard error of the mean.

Previous studies have shown that *Sle1a* induces T cell activation and results in loss of tolerance to chromatin (23, 26, 34). Furthermore, it has been reported that loss of tolerance and production of anti-chromatin IgG in B6.*Sle1a* mice displays a modest but not statistically significant female sex bias (23). However, it is not known if there is any sex bias in the T cell hyperactivation phenotype in B6.*Sle1a* mice. To test the hypothesis that the T cell hyperactivation phenotype shows a female sex bias in B6.*Sle1a* mice and to further examine the putative sex bias in the loss of tolerance in these mice, we utilized flow cytometry to assess the expression of immune cell activation markers and ELISA to quantify serum anti-chromatin IgG autoantibodies in female and male B6.*Sle1a* mice. We observed that 38% of B6.*Sle1a* females and 33% of B6.*Sle1a* males had lost tolerance and developed serum IgG anti-chromatin autoantibodies (**Figure 1**). Although the proportion of female B6.*Sle1a* mice that displayed loss of tolerance was slightly greater than that in male B6.*Sle1a* mice, this difference was not significant (p= 0.728). The proportion of B6.*Sle1a* mice that showed loss of tolerance in this analysis was roughly comparable to, albeit slightly less than, what has been reported previously (23).

**Figure 2:**
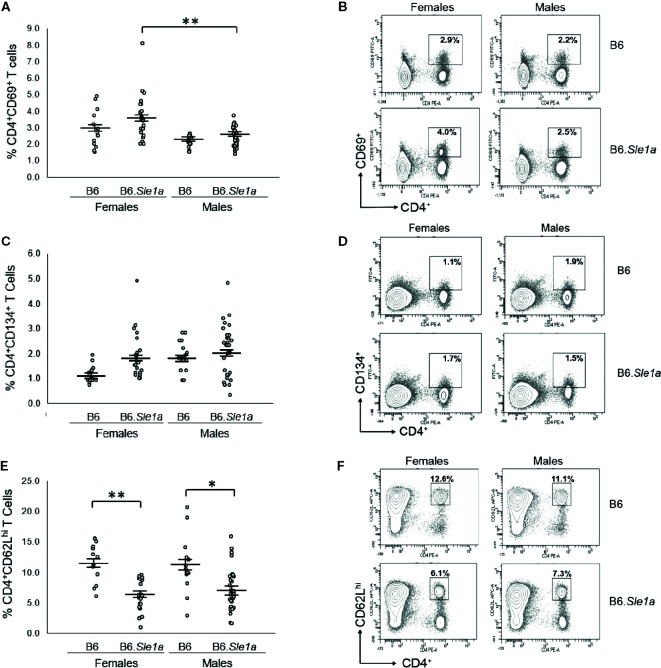
T cell hyperactivation shows a female sex bias in B6.*Sle1a* mice. **(A)** Dot plots show the percentage of splenocytes in female and male B6 and B6.*Sle1a* mice that were CD4^+^CD69^+^ activated T cells. **(B)** Representative contour plots from female and male B6 and B6.*Sle1a* mice show the frequency of CD4^+^CD69^+^ T cells. **(C)** Dot plots show the percentage of splenocytes in female and male B6 and B6.*Sle1a* mice that were CD4^+^CD134^+^ activated T cells. **(D)** Representative contour plots from female and male B6 and B6.*Sle1a* mice show the frequency of CD4^+^CD134^+^ T cells. **(E)** Dot plots show the percentage of splenocytes in female and male B6 and B6.*Sle1a* mice that were CD4^+^CD62L^hi^ T cells. **(F)** Representative contour plots from female and male B6 and B6.*Sle1a* mice show the frequency of CD4^+^CD62L^hi^ T cells. Splenocytes were collected from B6 female (N=14), B6.*Sle1a* female (N=24), B6 male (N=15), and B6.*Sle1a* male (N=30) mice that were 5–6 months of age. In **(A, C, E)**, the longer horizontal bar denotes the mean for each group, and the shorter black bars indicate the standard error of the mean. The * indicates p ≤ 0.05, and the ** indicates p ≤ 0.01.

Next, we examined the possibility that there is a sex bias in *Sle1a*-induced T cell hyperactivation. Although we observed trends toward increased proportions of CD4^+^ T cells expressing either the CD69 or CD134 activation markers in female B6.*Sle1a* compared to B6 controls, these differences did not achieve statistical significance (p=0.53; **Figures 2A, B** and p=0.20; **Figures 2C, D**). Nevertheless, compared to B6 controls, B6.*Sle1a* females did show a significant decrease in the proportion of splenic CD4^+^ T cells expressing high levels of the naïve T cell marker CD62L (**Figures 2E, F**). Notably, there was no evidence of any trend toward increased proportions of CD4^+^ T cells expressing either the CD69 or CD134 activation markers in B6.*Sle1a* male mice. A comparison of male B6 control and B6.*Sle1a* mice, revealed no significant differences in the proportion of activated CD4^+^ CD69^+^ T cells (p=1.00; **Figure 2A**) or CD4^+^ CD134^+^ T cells (p=0.99; **Figure 2C**). However, the proportion of CD4^+^ T cells expressing high levels of the naïve T cell marker CD62L in male B6.*Sle1a* mice was less than that in B6 controls (p ≤ 0.05; **Figure 2E**). A direct comparison of the T cell populations in the B6.*Sle1a* female and B6.*Sle1a* male mice, revealed that the proportion of CD4^+^CD69^+^ T cells in B6.*Sle1a* female mice was significantly greater than that in B6.*Sle1a* male mice (p ≤ 0.01; **Figure 2A**). This difference suggests that the T cell activation associated with *Sle1a* does show some degree of female sex bias despite the fact that no sex bias is observed in *Sle1a*-induced loss of tolerance. Interestingly, no significant difference was observed in the proportion of CD4^+^CD62L^hi^ T cells between B6.*Sle1a* female and B6.*Sle1a* male mice (p=0.97; **Figures 2E, F**). However, we note that the CD4^+^CD62L^hi^ population may include central memory T cells in addition to naïve T cells.

### ERα Deficiency Does Not Attenuate *Sle1a*-Induced T Cell Activation or Loss of Tolerance

**Figure 3:**
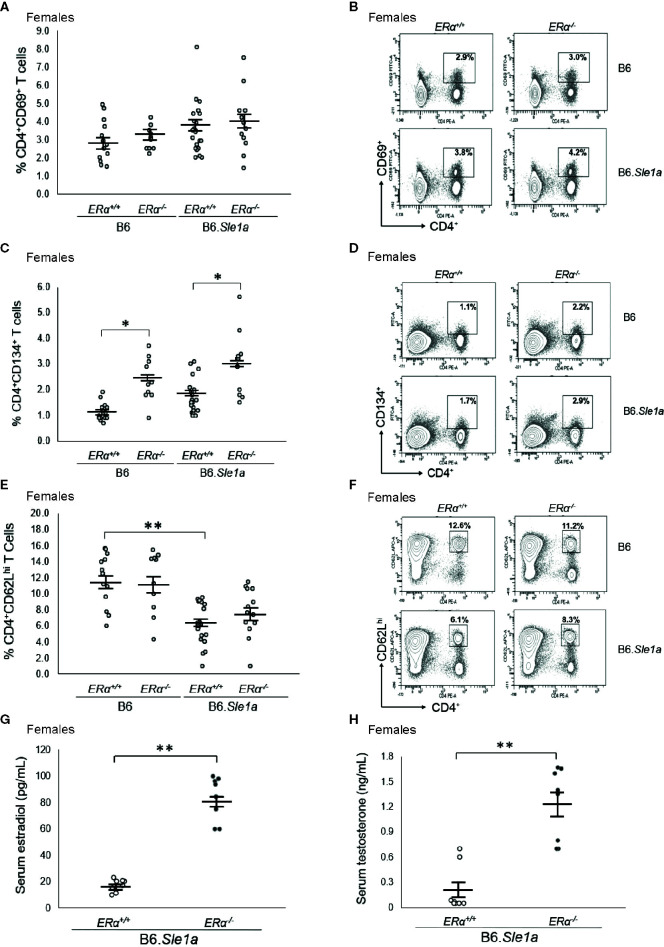
*ERα* deficiency does not affect T cell hyperactivation in female B6.*Sle1a* mice. **(A)** Dot plots show the percentage of splenocytes in female B6.*ERα^+/+^*, B6.*ERα^-/-^*, B6.*Sle1a*.*ERα^+/+^*, and B6.*Sle1a*.*ERα^-/-^* mice that were CD4^+^CD69^+^. **(B)** Representative contour plots from show the frequency of CD4^+^CD69^+^ T cells in female B6.*ERα^+/+^*, B6.*ERα^-/-^*, B6.*Sle1a*.*ERα^+/+^*, and B6.*Sle1a*.*ERα^-/-^* mice. **(C)** Dot plots show the percentage of splenocytes in female B6.*ERα^+/+^*, B6.*ERα^-/-^*, B6.*Sle1a*.*ERα^+/+^*, and B6.*Sle1a*.*ERα^-/-^* mice that were CD4^+^C62L^hi^ T cells. **(D)** Representative contour plots show the frequency of CD4^+^CD62L^hi^ T cells in female B6.*ERα^+/+^*, B6.*ERα^-/-^*, B6.*Sle1a*.*ERα^+/+^*, and B6.*Sle1a*.*ERα^-/-^* mice. **(E)** Dot plots show the percentage of splenocytes in female B6.*ERα^+/+^*, B6.*ERα^-/-^*, B6.*Sle1a*.*ERα^+/+^*, and B6.*Sle1a*.*ERα^-/-^* mice that were CD4^+^CD134^+^ activated T cells. **(F)** Representative contour plots show the frequency of CD4^+^CD134^+^ T cells in female B6.*ERα^+/+^*, B6.*ERα^-/-^*, B6.*Sle1a*.*ERα^+/+^*, and B6.*Sle1a*.*ERα^-/-^* mice. Splenocytes were collected from B6.*ERα^+/+^* (N=14), B6.*ERα^-/-^* (N=10), B6.*Sle1a*.*ERα^+/+^* (N=16), and B6.*Sle1a*.*ERα^-/-^* (N=13), female mice that were 5–6 months of age. **(G)** The concentration of total serum estradiol in female *ERα^+/+^* (N=8) and *ERα^-/-^* (N=8) B6.*Sle1a* congenic mice that were 5–6 months of age is shown. **(H)** The concentration of total serum testosterone in female *ERα^+/+^* (N=8) and *ERα^-/-^* (N=8) B6.*Sle1a* congenic mice that were 5–6 months of age is shown. In **(A, C, E, G, H)**, the longer horizontal bar denotes the mean for each group, and the shorter black bars indicate the standard error of the mean. The * indicates p ≤ 0.05, and the ** indicates p ≤ 0.01.

To test the hypothesis that the female sex bias in *Sle1a*-induced T cell activation is dependent upon ERα signaling, we intercrossed B6.*ERα* knockout mice with B6.*Sle1a* congenic mice to produce wild-type (*ERα^+/+^*), heterozygous (*ERα^+/-^*), and homozygous null (*ERα^-/-^*) mice and quantified the expression of immune cell activation markers and the levels of serum anti-chromatin IgG autoantibodies. No significant differences were observed between B6.*Sle1a*.*ERα^+/+^* and B6.*Sle1a*.*ERα^-/-^* female mice, with regard to the proportion of CD4^+^ T cells that express the activation marker CD69 (p=0.96; **Figures 3A, B**). Furthermore, contrary to what would be predicted if ERα were to promote *Sle1a*-induced T cell activation, the proportion of activated CD4^+^CD134^+^ T cells in B6.*Sle1a*.*ERα^-/-^* female mice was significantly greater than that seen in B6.*Sle1a*.*ERα^+/+^* females (p ≤ 0.05; **Figures 3C, D**). The proportion of CD4^+^CD62L^hi^ naïve T cells in B6.*Sle1a*.*ERα^-/-^* female mice did not differ significantly from that in B6.*Sle1a*.*ERα^+/+^* females (p=0.92; **Figures 3E, F**). These data indicate that *ERα* deficiency does not attenuate *Sle1a*-induced T cell activation in females.

Previous studies have shown that targeted disruption of ERα in mice causes perturbation of the hypothalamic-pituitary-gonadal axis that regulates estrogen biosynthesis leading to increased serum concentrations of estradiol and testosterone in *ERα^-/-^* female mice (35, 36). We previously observed this same disruption in of the hypothalamic-pituitary-gonadal axis in B6.*Sle1*.*ERα^-/-^* female mice (24). Likewise, we found that mean serum concentrations of both estradiol and testosterone were significantly greater in B6.*Sle1a*.*ERα^-/-^* female mice than in B6.*Sle1a*.*ERα^+/+^* female mice (p ≤ 0.01; **Figures 3G, H**).

When we analyzed the impact of *ERα* deficiency in *Sle1a*-induced T cell activation in males, the results obtained were similar to those observed in females. No significant differences were observed in the proportion of CD4^+^CD69^+^ T cells (p=0.37; **Supplementary Figures 1A, B**), CD4^+^CD134^+^ T cells (p=0.99; **Supplementary Figures 1C, D**) or CD4^+^CD62L^hi^ T cells (p=1.0; **Supplementary Figures 1E, F**) in B6.*Sle1a*.*ERα^+/+^* and B6.*Sle1a*.*ERα^-/-^* male mice. Altogether, these results indicate that the subtle female sex bias in T cell activation that we observed in B6.*Sle1a* mice is not dependent on ERα signaling.

In accordance with previous studies showing that *Sle1a* does not enhance B cell activation (23), we found that the proportion of B220^+^CD86^+^ B cells in female B6.*Sle1a*.*ERα^+/+^* mice was not different than that in female B6.*ERα^+/+^* controls (**Supplementary Figures 2A, B**). In fact, in male B6.*Sle1a*.*ERα^+/+^* mice, the proportion of B220^+^CD86^+^ B cells was actually significantly less than that in male B6.*ERα^+/+^* controls (p ≤ 0.01; **Supplementary Figures 2C, D**) as well as that in female B6.*Sle1a*.*ERα^+/+^* mice (p ≤ 0.01; **Supplementary Figure 2**). The potential implications of the decrease in the proportion of activated B cells in B6.*Sle1a*.*ERα^+/+^* males are not clear. Consistent with these observations, *Sle1a* did not impact surface expression the B cell activation marker CD22 (**Supplementary Figures 2E, F**). *ERα* genotype had no impact on B cell activation in B6.*Sle1a* mice (**Supplementary Figure 2**). Neither *Sle1a* nor *ERα* genotype had an impact on the relative proportion of germinal center B cells (**Supplementary Figure 3**) in these mice.

Although we did not observe a significant female sex bias in *Sle1a*-induced loss of tolerance, we nevertheless explored the possibility that this phenotype would be altered by ERα deficiency. ERα deficiency had no significant impact on the proportion of B6.*Sle1a* females or males that developed anti-chromatin IgG autoantibodies (p=0.84 (females); p=0.28 (males); **Supplementary Figure 4**).

### Disruption of ERα Attenuates Loss of Tolerance in B6.*Sle1b* Females and Eliminates the Female Sex Bias in *Sle1b*-Induced Loss of Tolerance

**Figure 4:**
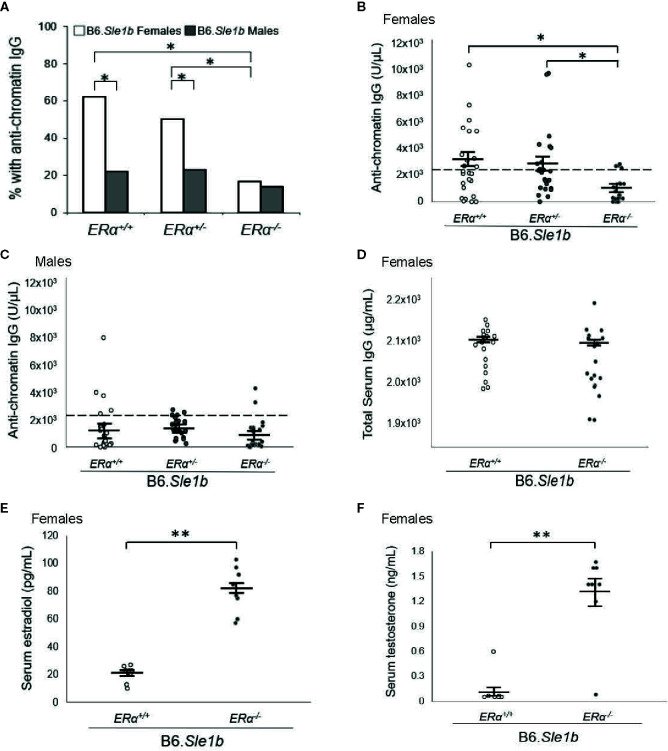
*ERα* disruption eliminates the female sex bias in *Sle1b*-induced loss of tolerance. **(A)** The proportion of B6.*Sle1b* female and male mice of all three ERα genotypes (*ERα^+/+^, ERα^+/-^, ERα^-/-^*) producing anti-chromatin IgG autoantibodies at 5 months of age is shown. **(B)** The concentration of anti-chromatin IgG autoantibodies in female *ERα^+/+^* (N=26)*, ERα^+/-^* (N=26), and *ERα^-/-^* (N=24) B6.*Sle1b* congenic mice is shown. **(C)** The concentration of anti-chromatin IgG autoantibodies in male *ERα^+/+^* (N=23)*, ERα^+/-^* (N=22), and *ERα^-/-^* (N=22) B6.*Sle1b* congenic mice is shown. In **(B)** and **(C)**, the dashed line represents the threshold used to designate a positive autoantibody titer in the experimental mice. This threshold was set at 2 standard deviations above the mean of a group of age-matched control B6 mice as has been described previously (23, 24). **(D)** The concentration of total serum IgG in female *ERα^+/+^* (N=18) and *ERα^-/-^* (N=18) B6.*Sle1b* congenic mice is shown. **(E)** The concentration of total serum estradiol in female *ERα^+/+^* (N=8) and *ERα^-/-^* (N=8) B6.*Sle1b* congenic mice that were 5–6 months of age is shown. **(F)** The concentration of total serum testosterone in female *ERα^+/+^* (N=8) and *ERα^-/-^* (N=8) B6.*Sle1b* congenic mice that were 5–6 months of age is shown. The longer black horizontal bar indicates the mean for each group, and the shorter black bars indicate the standard error of the mean. The * indicates p ≤ 0.05, and the ** indicates p ≤ 0.01.

Previous studies have shown that the *Sle1b* sublocus impacts loss of tolerance to chromatin and immune cell activation (23, 27, 30). Furthermore, both loss of tolerance and immune cell activation in B6.*Sle1b* congenic mice show a strong female sex bias (23, 30). To test the hypothesis that the female sex bias in the phenotypes observed in B6.*Sle1b* mice is dependent upon ERα signaling, we intercrossed B6.*ERα* knockout mice with B6.*Sle1b* congenic mice to produce *ERα^+/+^*, *ERα^+/-^*, and *ERα^-/-^* mice. In accordance with previously published studies, we observed that the proportion of B6.*Sle1b*.*ERα^+/+^* female mice that lost tolerance and developed anti-chromatin IgG autoantibodies was significantly greater than that in male B6.*Sle1b*.*ERα^+/+^* mice (62% versus 22%; p ≤ 0.05; **Figure 4A**). Heterozygosity for *ERα* did not have a significant impact on loss of tolerance in either B6.*Sle1b* females (62% versus 50%) or B6.*Sle1b* males (22% versus 23%) (**Figure 4A**). Strikingly, ERα deficiency in B6.*Sle1b* females dramatically attenuated loss of tolerance; The proportion of B6.*Sle1b.ERα^-/-^* female mice that developed anti-chromatin IgG autoantibodies was significantly less than that in B6.*Sle1b.ERα^+/+^* female mice (62% versus 17%; p ≤ 0.05; **Figures 4A, B**). Interestingly, the proportion of B6.*Sle1b.ERα^-/-^* female mice that developed anti-chromatin IgG autoantibodies was similar to that observed in B6.*Sle1b.ERα^+/+^* male mice (17% versus 22%; p≥0.05; **Figures 4A, B**). By contrast, ERα deficiency in male B6.*Sle1b* mice did not have a significant impact on loss of tolerance. The proportion of B6.*Sle1b.ERα^-/-^* male mice that developed anti-chromatin IgG did not differ from that in B6.*Sle1.ERα^+/+^* male mice (14% versus 22%; p=0.242 **Figures 4A, C**). These data indicate the female sex bias in *Sle1b*-induced loss of tolerance is dependent upon ERα signaling.

We have shown previously that the attenuated autoantibody development in ERα deficient B6.*Sle1* congenic mice and (NZB × NZW)F1 mice is not the result of a global defect in antibody production (12, 24). Nevertheless, to confirm that attenuated development of anti-chromatin IgG in B6.*Sle1b.ERα^-/-^* female mice was not due to a generic defect in antibody production, we examined the impact of *ERα* deficiency on total serum IgG in B6.*Sle1b* females. No significant difference in total serum IgG was observed between B6.*Sle1.ERα^+/+^* female mice and B6.*Sle1b.ERα^-/-^* female mice (2102 µg/ml versus 2073 µg/ml; p=0.14; **Figure 4D**). These data are consistent with the interpretation that the effect of ERα deficiency on anti-chromatin IgG in B6.*Sle1b* mice is due to an impact of ERα deficiency on the process of loss of tolerance and not the result of a broad defect in IgG production. As expected, ERα deficiency in B6.*Sle1b* females was associated with a significant increase in mean serum concentrations of estradiol (p ≤ 0.01; **Figure 4E**) and testosterone (p ≤ 0.01; **Figure 4F**).

### Disruption of *ERα* Abrogates *Sle1b*-Induced B Cell Hyperactivation in Females

**Figure 5:**
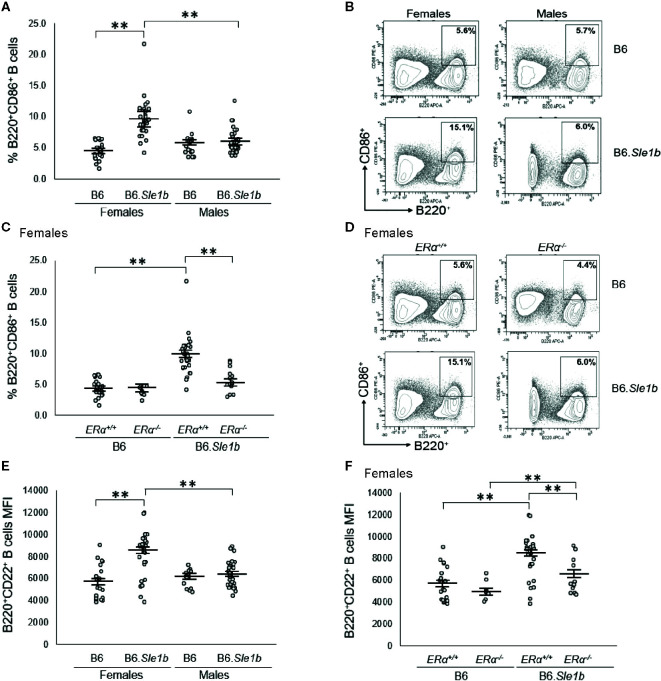
*ERα* disruption abrogates *Sle1b*-induced B cell hyperactivation in female mice. **(A)** Dot plots show the percentage of splenocytes that were B220^+^CD86^+^ activated B cells in female B6 (N=23), female B6.*Sle1b* (N =30), male B6 (N=19), and male B6.*Sle1b* (N= 32) mice. **(B)** Representative contour plots show the frequency of B220^+^CD86^+^ activated B cells in female and male B6 and B6.*Sle1b* mice. **(C)** Dot plots show the percentage of splenocytes that are B220^+^CD86^+^ activated B cells in female B6.*ERα^+/+^* (N=23), B6.*ERα^-/-^* (N=10), B6.*Sle1b*.*ERα^+/+^* (N=30), and B6.*Sle1b*.*ERα^-/-^* (N=13) mice. **(D)** Representative contour plots show the frequency of B220^+^CD86^+^ activated B cells in *ERα^+/+^, ERα^+/-^*, and *ERα^-/-^* B6.*Sle1b* female mice. **(E)** Dot plots show CD22 surface expression measured as mean fluorescence intensity (MFI) in B220^+^CD22^+^ B cells in female B6 (N=19), female B6.*Sle1b* (N=26), male B6 (N=14), and male B6.*Sle1b* (N=25) mice. **(F)** CD22 surface expression measured as mean fluorescence intensity (MFI) in B220^+^CD22^+^ B cells in female B6.*ERα^+/+^* (N=19), B6.*ERα^-/-^* (N=7), B6.*Sle1b*.*ERα^+/+^* (N=26), and B6.*Sle1b*.*ERα^-/-^* (N=13) mice is shown. Splenocytes were collected from mice that were 5–6 months of age. The longer horizontal bar in each panel denotes the mean for each group, and the shorter black bars indicate the standard error of the mean. The ** indicates p ≤ 0.01.

*Sle1b* is associated with a robust B cell hyperactivation phenotype that is observed in females only (30). Consistent with this previous report, we observed that the proportion of B220^+^ B cells expressing the activation marker CD86^+^ in B6.*Sle1b*.*ERα^+/+^* females was significantly greater than that in either B6.*ERα^+/+^* females or B6.*Sle1b*.*ERα^+/+^* males (p ≤ 0.01; **Figures 5A, B** **)**. To determine if this female-specific action of *Sle1b* is influenced by ERα signaling, we examined the impact of ERα deficiency on B cell activation in B6.*Sle1b* mice. In B6.*Sle1b*.*ERα^-/-^* females, the proportion of B220^+^ CD86^+^ activated B cells was significantly less than that in B6.*Sle1b*.*ERα^+/+^* females (p ≤ 0.01; **Figures 5C, D** **)**. Interestingly, the proportion of B220^+^ CD86^+^ B cells in B6.*Sle1b*.*ERα^-/-^* females was not significantly different than that in either B6.*ERα^-/-^* females (p=0.93; **Figures 5C, D** **)** or B6.*Sle1b*.*ERα^+/+^* males (p=1.0; **Figures 5A–D**). These data suggest that disruption of ERα abrogates *Sle1b*-induced B cell hyperactivation in B6.*Sle1b* females. The proportion of B220^+^CD86^+^ cells in male B6.*Sle1b*.*ERα^-/-^* mice was not different than that in B6.*Sle1b*.*ERα^+/+^* male mice (p=0.96; **Supplementary Figures 5A, B**) indicating that ERα deficiency had no impact on B cell activation in B6.*Sle1b* males.

We confirmed the results regarding the impact of ERα deficiency on *Sle1b*-induced B cell hyperactivation in females by examining a second B cell activation marker, CD22. We were also interested in examining CD22 because previous studies have suggested that continuous treatment of mice with supraphysiological levels of estrogens was associated with an increase in the expression of CD22 (37). In B220^+^ B cells from B6.*Sle1b*.*ERα^+/+^* females, surface expression of CD22, as measured by mean fluorescent intensity (MFI), was significantly greater than that in B220^+^ B cells from B6.*ERα^+/+^* females (8166 versus 5670; p ≤ 0.01: **Figure 5E**). These data indicate that in females, *Sle1b* increases B cell activation, as measured by CD22 MFI. CD22 MFI in B220^+^ B cells in B6.*Sle1b*.*ERα^+/+^* males was significantly less than that in B6.*Sle1b*.*ERα^+/+^* females (6383 versus 8166; p ≤ 0.01: **Figure 5E**) indicating that B cell activation as measured by CD22 MFI also displays a female sex bias in B6.*Sle1b* mice. Disruption of ERα eliminated the female sex bias in B cell activation as measured by CD22 MFI in B6.*Sle1b* mice. The CD22 MFI in B220^+^ B cells from B6.*Sle1b*.*ERα^-/-^* females was significantly less than that in B6.*Sle1b*.*ERα^+/+^* females (6266 versus 8166; p ≤ 0.01; **Figure 5F**) but was not different than that in B6.*Sle1b*.*ERα^+/+^* males (6266 versus 6383; p=1.0). Altogether, these data indicate that disruption of ERα abrogates *Sle1b*-induced B cell hyperactivation in B6.*Sle1b* females and suggest that ERα signaling is necessary for the B cell hyperactivation phenotype observed in B6.*Sle1b* females. Neither *Sle1b* nor *ERα* had any significant impact on CD22 MFI in males (**Figure 5E** and **Supplementary Figure 5C**).

### Disruption of ERα Attenuates *Sle1b*-Induced Spontaneous Germinal Center Formation in Females and Eliminates the Female Sex Bias in This Phenotype

**Figure 6:**
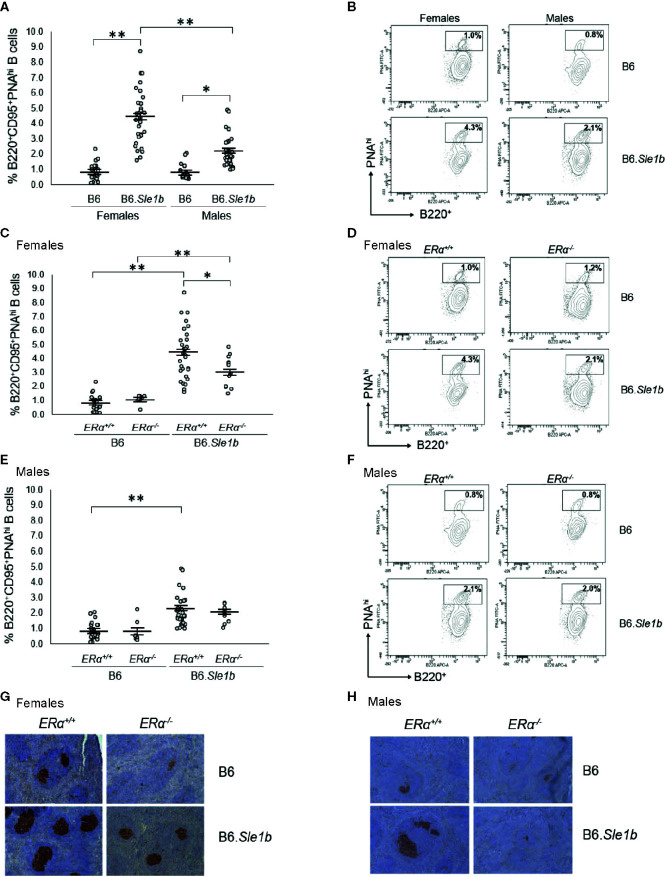
Disruption of *ERα* attenuates formation of spontaneous germinal center formation. **(A)** Dot plots show the percentage of splenocytes that were B220^+^CD95^+^PNA^hi^ germinal center B cells in female B6 (N=23), female B6.*Sle1b* (N =30), male B6 (N=19), and male B6.*Sle1b* (N= 32) mice. **(B)** Representative contour plots show the frequency of B220^+^CD95^+^PNA^hi^ B cells in female and male B6 and B6.*Sle1b* mice. **(C)** Dot plots show the percentage of splenocytes that were B220^+^CD95^+^PNA^hi^ germinal center B cells in female B6.*ERα^+/+^* (N=23), B6.*ERα^-/-^* (N=10), B6.*Sle1b*.*ERα^+/+^* (N=30), and B6.*Sle1b*.*ERα^-/-^* (N=13) mice. **(D)** Representative contour plots show the frequency of B220^+^ CD95^+^PNA^hi^ B cells in female B6.*ERα^+/+^*, B6.*ERα^-/-^*, B6.*Sle1b*.*ERα^+/+^*, and B6.*Sle1b*.*ERα^-/-^* mice. **(E)** Dot plots show the percentage of splenocytes that were B220^+^CD95^+^PNA^hi^ germinal center B cells in male B6.*ERα^+/+^* (N=19), B6.*ERα^-/-^* (N=7), B6.*Sle1b*.*ERα^+/+^* (N=32), and B6.*Sle1b*.*ERα^-/-^* (N=11) mice. **(F)** Representative contour plots show the frequency of B220^+^CD95^+^PNA^hi^ B cells in male B6.*ERα^+/+^*, B6.*ERα^-/-^*, B6.*Sle1b*.*ERα^+/+^*, and B6.*Sle1b*.*ERα^-/-^* mice. **(G)** Representative images of PNA staining in sections from the spleen of female B6.*ERα^+/+^* (N=3), B6.*ERα^-/-^* (N=3), B6.*Sle1b*.*ERα^+/+^* (N=7), and B6.*Sle1b*.*ERα^-/-^* (N=7) mice are shown. **(H)** Representative images of PNA staining in sections from the spleen of male B6.*ERα^+/+^*(N=3), B6.*ERα^-/-^* (N=3), B6.*Sle1b*.*ERα^+/+^* (N=7), and B6.*Sle1b*.*ERα^-/-^* (N=3) mice are shown. Spleens and splenocytes were collected from mice that were 5–6 months of age. The longer horizontal bar in each panel denotes the mean for each group, and the shorter black bars indicate the standard error of the mean. The * indicates p ≤ 0.05, and the ** indicates p ≤ 0.01.

In addition to having a greater proportion of activated B cells, the spleens of B6.*Sle1b* mice show enhanced formation of spontaneous germinal centers and an increase in the proportion of germinal center B cells (30, 31). Although both female and male B6.*Sle1b* mice exhibit spontaneous splenic germinal center formation, this phenotype has also been reported to show a significant female sex bias (30). Consistent with these results, we found a significantly greater proportion of B220^+^CD95^+^PNA^hi^ splenic germinal center B cells in B6.*Sle1b*.*ERα^+/+^* females and males than in sex-matched B6.*ERα^+/+^* mice (**Figures 6A, B**). Importantly, we also found that the proportion of B220^+^CD95^+^PNA^hi^ splenic germinal center B cells was significantly greater in B6.*Sle1b*.*ERα^+/+ ^*females than in B6.*Sle1b*.*ERα^+/+^* males (p ≤ 0.01; **Figures 6A, B**). Next, we examined the impact of ERα deficiency on germinal centers in B6.*Sle1b* mice using both flow cytometry and immunohistochemistry. Interestingly, ERα deficiency attenuated but did not completely abrogate expansion of the splenic germinal center B cells in B6.*Sle1b* females. Although the proportion of B220^+^CD95^+^PNA^hi^ splenic germinal center B cells in female B6.*Sle1b*.*ERα^-/-^* mice was significantly less than that in female B6.*Sle1b*.*ERα^+/+^* mice (p ≤ 0.01; **Figures 6C, D**), the proportion of B220^+^CD95^+^PNA^hi^ germinal center B cells in female B6.*Sle1b*.*ERα^-/-^* remained significantly greater than that in either B6.*ERα^+/+^* or B6.*ERα^-/-^* females (p ≤ 0.01; **Figures 6C, D**). Thus, the proportion of B220^+^CD95^+^PNA^hi^ germinal center B cells in female B6.*Sle1b*.*ERα^-/-^* mice was intermediate to that in female B6.*Sle1b*.*ERα^+/+^* mice and B6.*ERα^+/+^* females and was significantly different than both of these two groups of *ERα^+/+^* female mice. These data indicate that ERα deficiency attenuates but does not completely abrogate the ability of *Sle1b* to promote bypass of the germinal center checkpoint in females. Consistent with this interpretation, the proportion of B220^+^CD95^+^PNA^hi^ germinal center B cells in B6.*Sle1b*.*ERα^-/-^* female mice was not significantly different from that in B6.*Sle1b*.*ERα^+/+^* male mice (p= 0.133; **Figures 6A–D**). Disruption of *ERα* did not have a significant impact on the germinal center B cell checkpoint in B6.*Sle1b* male mice; The proportion of B220^+^CD95^+^PNA^hi^ germinal B cells in B6.*Sle1b*.*ERα^-/-^* male mice did not differ significantly from that in B6.*Sle1b*.*ERα^+/+^* male mice (p=1.0: **Figures 6E, F**). Altogether, these data indicate that ERα deficiency attenuates spontaneous germinal center formation in B6.*Sle1b* congenic females and eliminates the female sex bias that is observed in this phenotype in B6.*Sle1b* mice.

The ability of ERα deficiency to attenuate bypass of the germinal center B cell checkpoint and expansion of the germinal center B cell population in B6.*Sle1b* females was also assessed using immunohistochemical staining. For this analysis, germinal centers were identified in sections of spleens *via* PNA staining. Using this method, we observed that the number and size of splenic germinal centers in B6.*Sle1b* mice were significantly greater than in B6 controls, and these increases were much more prominent in B6.*Sle1b* females than males (**Figures 6G, H**). Disruption of ERα in female B6.*Sle1b* mice was associated with a reduction in the number and size of spontaneous germinal centers (**Figure 6G**). By contrast, ERα deficiency did not appear to have a significant impact on spontaneous germinal center formation in B6.*Sle1b* male mice (**Figure 6H**). These data support the interpretation that ERα signaling is responsible for the sex bias in *Sle1b*-induced spontaneous germinal center formation.

**Figure 7:**
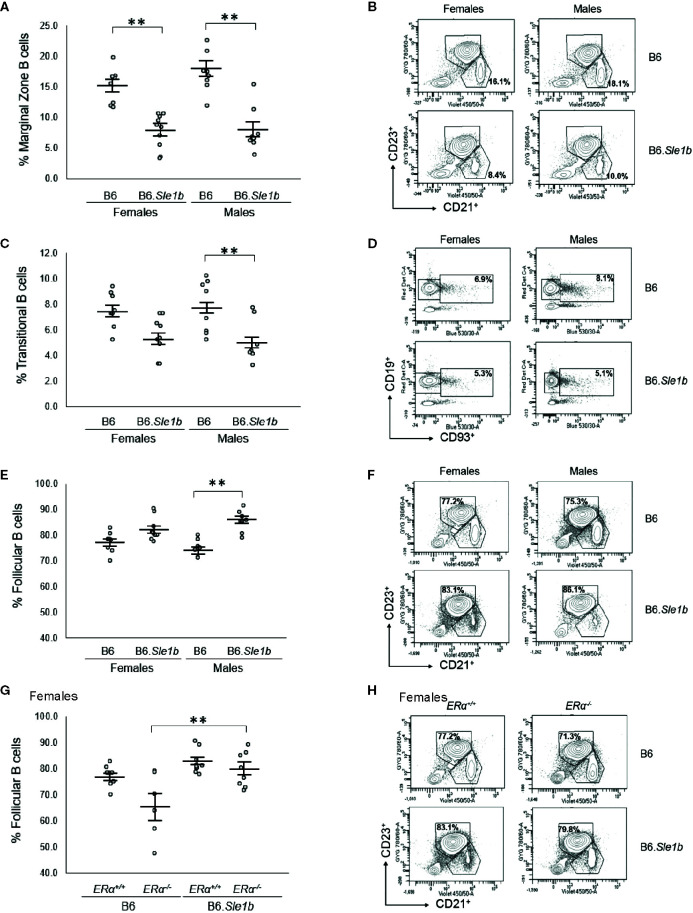
*Sle1b* is associated with a decrease in the marginal zone B cell population, but this effect is not modulated by ERα in female B6.*Sle1b* mice. **(A)** Dot plots show the percentage of splenic marginal zone B cells (identified as lymphocyte singlets that were CD5^-^CD19^+^CD93^-^CD21^+^CD23^-^) in female and male B6 and B6.*Sle1b* mice (N= 8 per group). **(B)** Representative contour plots show the frequency of marginal zone B cells in female and male B6 and B6.*Sle1b* mice. **(C)** Dot plots show the percentage of splenic transitional B cells (identified as lymphocyte singlets that were CD5^-^CD19^+^CD93^+^) in female and male B6 and B6.*Sle1b* mice (N= 8 per group). **(D)** Representative contour plots show the frequency of splenic transitional B cells in female and male B6 and B6.*Sle1b* mice. **(E)** Dot plots show the percentage of splenic follicular B cells (identified as lymphocyte singlets that were CD5^-^CD19^+^CD93^-^CD21^-^CD23^+^) in female and male B6 and B6.*Sle1b* mice (N= 8 per group). **(F)** Representative contour plots show the frequency of follicular B cells in female and male B6 and B6.*Sle1b* mice. **(G)** Dot plots show the percentage of follicular B cells in female B6.*ERα^+/+^* (N=8), B6.*ERα^-/-^* (N=6), B6.*Sle1b*.*ERα^+/+^* (N=8), and B6.*Sle1b*.*ERα^-/-^* mice (N=8). **(H)** Representative contour plots show the frequency of follicular B cells in female B6.*ERα^+/+^*, B6.*ERα^-/-^*, B6.*Sle1b*.*ERα^+/+^*, and B6.*Sle1b*.*ERα^-/-^* mice. Splenocytes were collected from mice that were 5–6 months of age. The longer horizontal bar in each panel denotes the mean for each group, and the shorter black bars indicate the standard error of the mean. The ** indicates p ≤ 0.01.

In addition to showing an expansion of the germinal center B cell population, the spleens of B6.*Sle1b* female mice have been reported to display a decrease in relative abundance of marginal zone B cells (38). However, it is not known if this phenotype displays a sex bias. Our analysis revealed that the spleens of both female and male B6.*Sle1b* mice exhibit reduced proportions of marginal zone B cells compared to sex-match B6 mice (p ≤ 0.01; **Figures 7A, B**). There was no evidence of a sex bias in this phenotype, and ERα deficiency did not significantly alter the impact of *Sle1b* on the marginal zone B cell population (**Figures 7A, B** and **Supplementary Figure 6**).

Although B6.*Sle1b* females have a decrease in relative abundance of splenic marginal zone B cells, it was reported that these mice exhibit no significant increase in the proportion of follicular B cells (38). Consistent with this observation, we observed no significant changes in the relative abundance of either follicular B cells or transitional B cells in the spleens of B6.*Sle1b* females (**Figures 7C–F**). Nevertheless, there seemed to be a trend toward a decrease in the frequency of transitional B cells and a trend toward an increase in the frequency of follicular B cells in B6.*Sle1b* females. By contrast, compared to spleens from B6 males, the spleens from B6.*Sle1b* males displayed a significantly decreased proportion of transitional B cells and increased proportion of follicular B cells (p ≤ 0.01; **Figures 7C–F**). As noted previously, similar trends, though evident in the B6.*Sle1b* females, did not achieve statistical significance. Although *ERα* deficiency did not impact the relative proportion of follicular B cells in B6.*Sle1b* females, we did note that B6.*Sle1bERα^-/-^* females also showed an expansion in the splenic follicular B cell population compared to B6.*ERα^-/-^* females (p ≤ 0.01; **Figures 7G, H**). ERα deficiency did not attenuate the expansion of the follicular B cell subset in B6.*Sle1b* males (**Supplementary Figures 7A, B**). Likewise, ERα had no impact on the relative abundance of the transitional B cell population in B6.*Sle1b* congenic females or males (**Supplementary Figures 7C–F**).

### Disruption of *ERα* Attenuates T Cell Hyperactivation in B6.*Sle1b* Females and Eliminates the Female Sex Bias in *Sle1b*-Induced T Cell Activation

**Figure 8:**
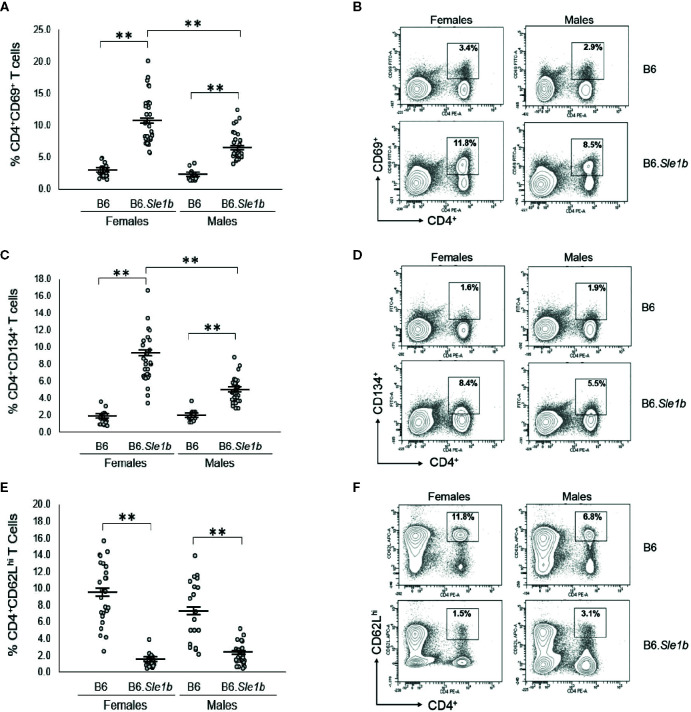
T cell hyperactivation in B6.*Sle1b* displays a female sex bias. **(A)** Dot plots show the percentage of splenocytes in female and male B6 and B6.*Sle1b* mice that were CD4^+^CD69^+^ activated T cells. **(B)** Representative contour plots from female and male B6 and B6.*Sle1b* mice show the frequency of CD4^+^CD69^+^ T cells. **(C)** Dot plots show the percentage of splenocytes in female and male B6 and B6.*Sle1b* mice that were CD4^+^C62L^hi^ T cells. **(D)** Representative contour plots from female and male B6 and B6.*Sle1b* mice show the frequency of CD4^+^CD62L^hi^ T cells. **(E)** Dot plots show the percentage of splenocytes in female and male B6 and B6.*Sle1b* mice that were CD4^+^CD134^+^ activated T cells. **(F)** Representative contour plots from female and male B6 and B6.*Sle1b* mice show the frequency of CD4^+^CD134^+^ T cells. Splenocytes were collected from B6 female (N=23), B6.*Sle1b* female (N=30), B6 male (N=19), and B6.*Sle1b* male (N=32) mice that were 5–6 months of age. In **(A, C, E)**, the longer horizontal bar denotes the mean for each group, and the shorter black bars indicate the standard error of the mean. The ** indicates p ≤ 0.01.

We previously showed that *ER*α deficiency attenuated but did not completely eliminate T cell hyperactivation in B6*.Sle1* females (24). The *Sle1b* sublocus contributes to the T cell activation phenotype in B6.*Sle1* mice, and the T cell activation in B6.*Sle1b* mice displays significant female sex bias (30). Thus, we sought to examine the impact of ERα deficiency on T cell hyperactivation in B6.*Sle1b* mice. As expected, B6.*Sle1b*.*ERα^+/+^* female mice showed robust T cell hyperactivation as evidenced by an increase in the proportions of CD4^+^CD69^+^ and CD4^+^CD134^+^ activated T cells and a decrease in the proportion of naïve CD4^+^CD62L^hi^ T cells when compared to that in B6.*ERα^+/+^* female mice (p ≤ 0.01; **Figure 8**). B6.*Sle1b*.*ERα^+/+^* male mice also exhibited an increase in the proportions of CD4^+^CD69^+^ and CD4^+^CD134^+^ activated T cells and a decrease in the proportion of naïve CD4^+^CD62L^hi^ T cells when compared to that in B6.*ERα^+/+^* male mice (p ≤ 0.01; **Figure 8**). Consistent with the previously described female sex bias in *Sle1b*-induced T cell activation, we found that the proportion of both CD4^+^CD69^+^ and CD4^+^CD134^+^ activated T cells in B6.*Sle1b*.*ERα^+/+^* female mice was significantly greater than that in B6.*Sle1b*.*ERα^+/+^* male mice (**Figures 8A–D**).

**Figure 9:**
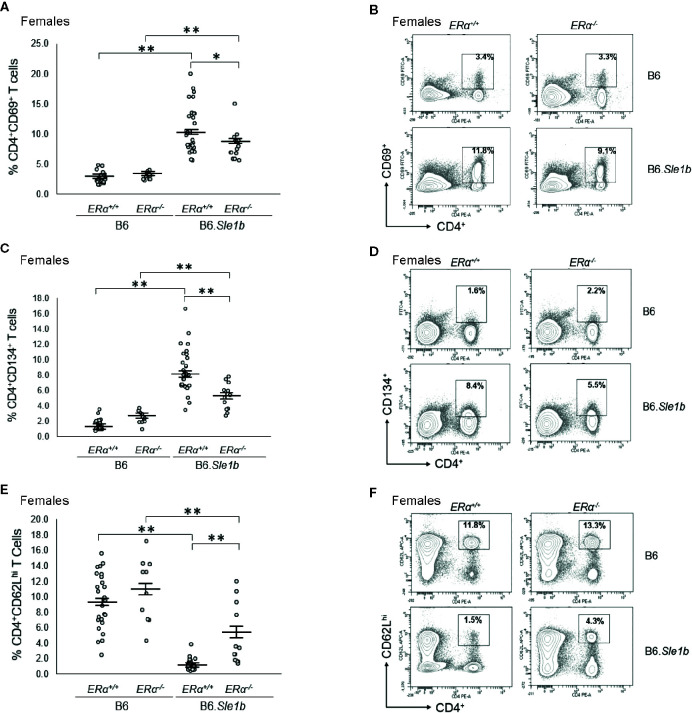
Disruption of ERα attenuates *Sle1b*-induced T cell hyperactivation in female mice. **(A)** Dot plots show the percentage of splenocytes in female B6.*ERα^+/+^*, B6.*ERα^-/-^*, B6.*Sle1b*.*ERα^+/+^*, and B6.*Sle1b*.*ERα^-/-^* mice that were CD4^+^CD69^+^ activated T cells. **(B)** Representative contour plots show the frequency of CD4^+^CD69^+^ T cells in female B6.*ERα^+/+^*, B6.*ERα^-/-^*, B6.*Sle1b*.*ERα^+/+^*, and B6.*Sle1b*.*ERα^-/-^* mice. **(C)** Dot plots show the percentage of splenocytes in female B6.*ERα^+/+^*, B6.*ERα^-/-^*, B6.*Sle1b*.*ERα^+/+^*, and B6.*Sle1b*.*ERα^-/-^* mice that were CD4^+^CD134^+^ T cells. **(D)** Representative contour plots show the frequency of CD4^+^CD134^+^ T cells in female B6.*ERα^+/+^*, B6.*ERα^-/-^*, B6.*Sle1b*.*ERα^+/+^*, and B6.*Sle1b*.*ERα^-/-^* mice. **(E)** Dot plots show the percentage of splenocytes in female B6.*ERα^+/+^*, B6.*ERα^-/-^*, B6.*Sle1b*.*ERα^+/+^*, and B6.*Sle1b*.*ERα^-/-^* mice that were CD4^+^CD62L^hi^ T cells. **(F)** Representative contour plots show the frequency of CD4^+^CD62L^hi^ cells in female B6.*ERα^+/+^*, B6.*ERα^-/-^*, B6.*Sle1b*.*ERα^+/+^*, and B6.*Sle1b*.*ERα^-/-^* mice. Splenocytes were collected from female B6.*ERα^+/+^* (N=23), B6.*ERα^-/-^* (N=10), B6.*Sle1b*.*ERα^+/+^* (N=30), and B6.*Sle1b*.*ERα^-/-^* (N=13) mice that were 5–6 months of age. In **(A), (C),** and **(E)**, the longer horizontal bar denotes the mean for each group, and the shorter black bars indicate the standard error of the mean. The * indicates p ≤ 0.05, and the ** indicates p ≤ 0.01.

When we examined the impact of ERα deficiency on T cell hyperactivation in B6.*Sle1b* females, we observed that T cell activation was reduced but not eliminated by disruption of *ERα*. In B6.*Sle1b*.*ERα^-/-^* females, the proportion of CD4^+^CD69^+^ and CD4^+^CD134^+^ activated T cells was significantly less than that B6.*Sle1b*.*ERα* ^+/+^ females (**Figures 9A–D**). However, the proportion of CD4^+^CD69^+^ and CD4^+^CD134^+^ activated T cells in B6.*Sle1b*.*ERα^-/-^* females remained significantly greater than that in B6.*ERα* ^+/+^ and B6.*ERα^-/-^* female mice (**Figures 9A–D**). Consistent with the observation that ERα deficiency partially attenuated T cell activation in B6.*Sle1b* females, we found that the proportion of CD4^+^ T cells expressing high levels of the naïve T cell marker CD62L was significantly greater in B6.*Sle1b*.*ERα^-/-^* female mice than that in B6.*Sle1b*.*ERα^+/+^* females (**Figures 9E, F**). However, the proportion of CD4^+^CD62L^hi^ T cells in B6.*Sle1b*.*ERα^-/-^* female mice remained significantly less than that in B6.*ERα* ^+/+^ and B6.*ERα^-/-^* female mice (**Figures 9E, F**). Overall, the proportions of CD4^+^CD69^+^ T cells, CD4^+^CD134^+^ T cells, and CD4^+^CD62Lhi^+^ T cells in B6.*Sle1b*.*ERα^-/-^* female mice did not differ significantly from that in B6.*Sle1b*.*ERα^+/+^* males (**Figures 8** and **9**). ERα deficiency had no impact on *Sle1b*-induced T cell activation in males (**Supplementary Figure 8**). Altogether, these results indicate that ERα deficiency partially attenuates *Sle1b*-induced T cell activation in females, and completely eliminates the sex bias in *Sle1b*-induced T cell activation. These data suggest that *Sle1b* induces T cell activation *via* two distinct processes, one that is ERα-dependent and another that is ERα-independent. The portion of *Sle1b*-induced T cell activation that is ERα-independent is that which is seen in B6.*Sle1b* males and remains in B6.*Sle1b*.*ERα^-/-^* female mice.

### Disruption of ERα Abrogates Female *Sle1b*-Induced T_fh_ Cell Hyperactivation

**Figure 10:**
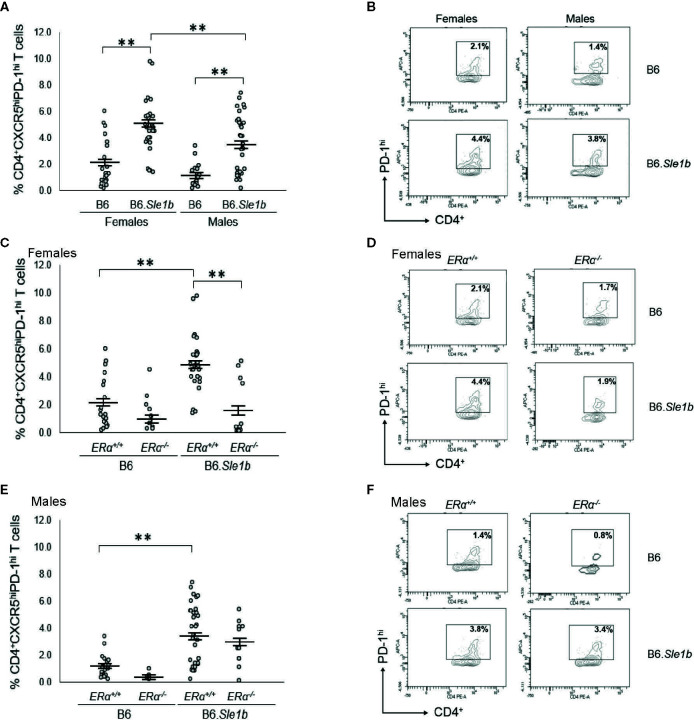
Disruption of ERα abrogates *Sle1b*-induced T_fh_ cell hyperactivation in female mice. **(A)** Dot plots show the percentage of splenocytes in female B6 (N=23), female B6.*Sle1b* (N =30), male B6 (N=19), and male B6.*Sle1b* (N= 32) mice that were CD4^+^CXCR5^hi^PD-1^hi^ follicular helper T (T_fh_) cells. **(B)** Representative contour plots show the frequency of CD4^+^CXCR5^hi^PD-1^hi^ T_fh_ cells in female and male B6 and B6.*Sle1b* mice. **(C)** Dot plots show the percentage of splenocytes in female B6.*ERα^+/+^* (N=23), B6.*ERα^-/-^* (N=10), B6.*Sle1b*.*ERα^+/+^* (N=30), and B6.*Sle1b*.*ERα^-/-^* (N=13) mice that were CD4^+^CXCR5^hi^PD-1^hi^ T_fh_ cells. **(D)** Representative contour plots show the frequency of CD4^+^CXCR5^hi^PD-1^hi^ T_fh_ cells in female B6.*ERα^+/+^*, B6.*ERα^-/-^*, B6.*Sle1b*.*ERα^+/+^*, and B6.*Sle1b*.*ERα^-/-^* mice. **(E)** Dot plots show the percentage of splenocytes in male B6.*ERα^+/+^* (N=19), B6.*ERα^-/-^* (N=5), B6.*Sle1b*.*ERα^+/+^*(N=32), and B6.*Sle1b*.*ERα^-/-^* (N=11) mice that were CD4^+^CXCR5^hi^PD-1^hi^ T_fh_ cells. **(F)** Representative contour plots show the frequency of CD4^+^CXCR5^hi^PD-1^hi^ T_fh_ cells in male B6.*ERα^+/+^*, B6.*ERα^-/-^*, B6.*Sle1b*.*ERα^+/+^*, and B6.*Sle1b*.*ERα^-/-^* mice. Splenocytes were collected from mice that were 5–6 months of age. In **(A, C, E)**, the longer horizontal bar in each panel denotes the mean for each group, and the shorter black bars indicate the standard error of the mean. The ** indicates p ≤ 0.01.

In addition to showing an increase in the proportion of CD4^+^CD69^+^ and CD4^+^CD134^+^ activated splenic T cells, B6.*Sle1b* mice also show an increase in the proportion of splenic CD4^+^CXCR5^hi^PD-1^hi^ T follicular helper cells (T_fh_ cells) (30). This increase in the T_fh_ cell population has been reported to show a female sex bias (30). To determine if the female sex bias in the expansion of the T_fh_ cell population in B6. *Sle1b* mice was also dependent, at least in part, on ERα, we examined the impact of ERα deficiency on this specific T cell subset. As has been reported previously, we observed that both female and male B6.*Sle1b.ERα^+/+^* mice showed a significantly greater proportion of splenic CD4^+^CXCR5^hi^PD-1^hi^ T cells than sex-matched B6.*ERα^+/+^* mice (**Figures 10A, B**). Furthermore, the proportion of T_fh_ cells in B6.*Sle1b.ERα^+/+^* females was greater than that in B6.*Sle1b.ERα^+/+^* males (p≤ 0.01; **Figures 10A, B**). ERα deficiency attenuated the expansion of the T_fh_ cell population in B6.*Sle1b* females; The proportion of CD4^+^CXCR5^hi^PD-1^hi^ T cells in B6.*Sle1b*.*ERα^-/-^* female mice was significantly less than that in B6.*Sle1b*.*ERα^+/+^* females (p≤0.01; **Figures 10C, D**). The proportion of CD4^+^CXCR5^hi^PD-1^hi^ T cells in B6.*Sle1b*.*ERα^-/-^* female mice did not differ significantly from that in B6.ER*α^+/+^* or B6.*ERα^-/-^* females (p =1.0; **Figures 10C, D**). ERα deficiency did not impact the proportion of CD4^+^CXCR5^hi^PD-1^hi^ T cells in B6.*Sle1b*.*ERα* male mice (**Figures 10E, F**) Overall, these data indicate that ERα deficiency eliminates the sex bias in *Sle1b*-induced expansion of the T_fh_ cell population, but does not completely abrogate the effect of *Sle1b* on this T cell subset. However, one caveat to this analysis is that CD4^+^CXCR5^hi^PD-1^hi^ T cell population may include exhausted T cells as well as T_fh_ cells.

## Discussion

It has been previously established that the lupus susceptibility locus *Sle1* shows a strong female sex bias, and we have shown that this female sex bias is mediated by estrogens working through ERα (23, 24). *Sle1* is composed of three subloci, each of which acts independently to promote loss of tolerance through distinct mechanisms (23). Although two of these *Sle1* subloci show at least some degree of female sex bias, it is not known to what extent ERα signaling modulates that actions of the individual *Sle1* subloci. Here, we show that ERα signaling enhances the action of *Sle1b*, the *Sle1* sublocus with the most robust impact on loss of tolerance, the most pronounced female sex bias, and the only sublocus that impacts B cell activation and germinal center formation. Indeed, some of the effects of *Sle1b* are not just female sex biased, but female sex specific, and these manifestations of *Sle1b* are completely abrogated by disruption of ERα, suggesting that these phenotypes require the synergistic actions of *Sle1b* and ERα signaling. By contrast, disruption of ERα had no impact on the actions of the *Sle1a* sublocus, which shows a modest female sex bias.

Consistent with what has been reported previously, we observed a slight but non-significant female sex bias in B6. *Sle1a*-induced loss of tolerance. Perhaps because of this fact, previous studies of B6.*Sle1a* mice have not mentioned examining the possibility that the ability of *Sle1a* to promote T cell activation might also show a female sex bias, so we examined this explicitly. Here we report for the first time that *Sle1a*-induced T cell hyperactivation shows a female sex bias. However, disruption of ERα did not impact *Sle1a*-induced T cell hyperactivation indicating that the female sex bias in this phenotype is not dependent upon ERα signaling. These results are consistent with our previous studies on the impact of sex and ERα on the expression of the *Pbx-1* isoforms that underlie *Sle1a*; We showed that although T cells from female B6.*Sle1* mice express higher levels of some *Pbx-1* isoforms than males, this female sex bias is not attenuated by disruption of ERα (24). The observation that the female sex bias in *Sle1a*-induced T cell activation and expression of the gene underlying *Sle1a* is not impacted by disruption of ERα underscores the importance of not making the assumption that a female sex bias in a given phenotype reflects the actions of estrogens or estrogen receptor signaling. The female sex bias in the *Sle1a*-induced T cell hyperactivation phenotype could, for example, be due to the presence of two X chromosomes in females. Indeed, studies have reported abnormalities in X chromosome inactivation and upregulation in the expression of some X-linked genes in the T cells of women with lupus and/or lupus-prone mice (39, 40).

The *Sle1b* sublocus has a very pronounced female sex bias in loss of tolerance, B and T cell hyperactivation, and spontaneous germinal center formation (30). Indeed, one of the phenotypes associated with *Sle1b*, B cell hyperactivation, is female specific (30, 31). We found that the ability of *Sle1b* to promote B cell hyperactivation exclusively in female mice is dependent upon ERα; Disruption of ERα completely eliminated B cell hyperactivation in B6.*Sle1b* females. These results are consistent with our previous work showing that B cell hyperactivation in B6.*Sle1* mice was likewise female specific and fully dependent upon ERα signaling. The observation that neither ERα nor *Sle1b* alone induces B cell hyperactivation in females indicates that the female specific B cell hyperactivation phenotype in B6.*Sle1b* mice requires synergy between ERα signaling and *Sle1b*. We have previously shown that neither sex nor ERα genotype impact the expression of *CD48* or *Ly10*8 isoforms, which underlie the *Sle1b* sublocus (24). These data indicate that the molecular basis for this synergy is not simply ERα-dependent regulation of genes underlying the *Sle1b* sublocus.

The *Sle1b* locus contains members of the signaling lymphocyte activation molecules (SLAM) gene family, which modulate antigen receptor signaling, immune cell activation, and differentiation (27). These genes are expressed in and modulate activation and tolerance in both B and T cells. The B cell intrinsic actions of the *Sle1b* locus as a whole as well as some individual SLAM family members contained within *Sle1b*, such as Ly108 and CD84, have been shown to modulate BCR signaling and to promote bypass of the germinal center B cell checkpoint, the formation of spontaneous germinal centers, and the development of anti-chromatin autoantibodies in B6.*Sle1b* congenic mice (30, 31). Likewise, we have shown that the ability ERα to promote loss of tolerance and autoantibody production in the (NZB × NZW)F1 lupus model involves B cell intrinsic action of ERα (13). Estrogens, acting through ERα have also been suggested to modulate BCR signaling (41). These previous studies, together with our observation that the B cell hyperactivation phenotype in B6.*Sle1b* females is completely abrogated by disruption of ERα, suggest that coordinated regulation of BCR signaling and B cell activation may, at least in part, be required for the molecular synergy between *Sle1b* and ERα.

Most of the phenotypes associated with *Sle1b*, including loss of tolerance/development of anti-chromatin IgG, spontaneous formation of germinal centers, and T cell hyperactivation show a strong female sex bias but are not female specific. Disruption of ERα in B6.*Sle1b* females attenuated all of these female sex biased phenotypes. Furthermore, these phenotypes in B6.*Sle1b*.*ERα^-/-^* female mice were quantitatively indistinguishable from those observed in B6.*Sle1b* males, indicating that ERα was responsible for the female sex bias observed for each of these phenotypes. The residual loss of tolerance/development of anti-chromatin IgG, spontaneous formation of germinal centers, and T cell hyperactivation observed in B6.*Sle1b*.*ERα^-/-^* female mice and present in B6.*Sle1b* males likely reflects the activity of *Sle1b* in the absence of the robust ERα signaling that is seen in *ERα^+/+^* females. These results indicate that in the absence of strong ERα signaling, *Sle1b* induces some loss of tolerance, expansion of the germinal center B cell population, and T cell hyperactivation. ERα augments these effects of *Sle1b* exclusively in females, suggesting that the higher level of ERα signaling in females is required for this enhancement. Thus, the ability of *Sle1b* to induce loss of tolerance/development of anti-chromatin IgG, spontaneous formation of germinal centers, and T cell hyperactivation, is potentiated by but not dependent upon ERα signaling.

It has been reported previously that *Sle1b* is associated with a reduction in the relative abundance of marginal zone B cells in female mice. Here, we report that this phenotype is also present in B6.*Sle1b* males, and there is no evidence of a female sex bias in this reduction in the marginal zone B cell population. We also observed that *Sle1b* was also associated with a decrease in the relative abundance of the transitional B cell population and an increase in a relative abundance of the follicular B cell subset, but these trends only reached statistical significance in male mice. The observation that *Sle1b* induced a more dramatic increase in the follicular B cell subset in males was interesting given the fact that *Sle1b* was also associated with expansion of the germinal center B cell population in both females and males. In this regard, we note that the expansion of the germinal center B cell population was more dramatic in females than males, and this sex bias was dependent upon ERα. Altogether, these data may suggest that *Sle1b* promotes development of follicular B cells from transitional B cells and the development of germinal center B cells from follicular B cells in both females and males. However, robust ERα signaling in females may also independently promote the development of the germinal center B cells from follicular B cells in B6.*Sle1b* females, resulting in a larger proportion of follicular B cells becoming germinal center B cells. Thus, the result of these combined effects of *Sle1b* and ERα would be a more dramatic expansion of the germinal center B cell population in B6.*Sle1b* females compared to males, and a decrease in size of the follicular B cell population in B6.*Sle1b* females compared to males.

It has also been reported that continuous treatment with supraphysiological levels of estrogens results in an expansion of the marginal zone B cell subset (37). However, Hill et al. reported that the ability estrogens to modulate the marginal zone B cell population is independent of both ERα and ERβ (41). If physiological levels of estrogens likewise promoted expansion of the marginal zone B cell population, and this phenomenon required ERα, then we would anticipate that disruption of ERα might lead to a reduction in the marginal zone B cell population. As mentioned previously, we found that *Sle1b* itself was associated with a decrease in abundance of the marginal zone population, and we observed no sex bias in this phenotype. Disruption of ERα did not significantly decrease the relative abundance of the marginal zone B cell subset in B6.*Sle1b* mice. In fact, we found that ERα deficiency was associated with a trend, albeit not a statistically significant one, toward an increased frequency of marginal zone B cells. This latter observation is consistent with our previous work showing that B cell specific deletion of ERα and the resulting attenuation of lupus in (NZB × NZW)F1 mice is associated with an expansion of the marginal zone B cell population (13). Thus, our results are consistent with those of Hill et al., and suggest that ERα signaling does not promote development of marginal zone B cells.

In the current study, we used a targeted knockout allele of ERα that results in global disruption of the classical, full length 66 kilodalton ERα protein (32, 35). As a consequence of this ERα knockout, all biological processes that require full length ERα, such as development of ovarian follicles and mammary glands, fertility, and the functioning of the hypothalamic-pituitary-gonadal axis, which regulates estrogen biosynthesis, are disrupted (32, 35, 36). As a result, *ERα^-/-^* female mice, which are homozygous for this ERα knockout allele, have serum estradiol levels that are significantly higher than cycling *ER^+/+^* female mice and serum testosterone levels equivalent to that in intact *ER^+/+^* male mice (35, 36). Consistent with these previous studies, we also observed that this *ERα* knockout was associated with increased serum concentrations of estradiol and testosterone in B6.*Sle1a* and B6.*Sle1b* female mice. It has been postulated that the ability of this *ERα* knockout allele to attenuate loss of tolerance, autoantibody production, and development of lupus might be due, at least in part, to the increased levels of androgens, which can reduce autoantibodies and lupus pathogenesis (42, 43). Although we have not explicitly examined this possibility here, in a previous study, we found that in B6.*Sle1* female mice, the impact of ovariectomy on loss of tolerance and autoantibody development was equivalent to the impact of this *ERα* knockout allele on these parameters (24). Furthermore, in this previous study we observed no correlation between serum levels of testosterone and autoantibodies in intact and ovariectomized *B6.Sle1* mice of various *ERα* genotypes (24). These data indicated that it is disruption of estrogen signaling *via* full length ERα and not increased androgen levels that resulted in the attenuation of the autoimmune phenotype in B6.*Sle1*.*ERα^-/-^* females. Consistent with this interpretation, we previously reported that in lupus prone (NZB x NZW)F1 females, B cell specific deletion of *ERα*, which does not perturb the hypothalamic-pituitary-gonadal axis or alter serum levels of estrogens or androgens, nevertheless attenuates development of autoatibodies, immune cell activation, and lupus nephritis (13).

Even though the ERα knockout allele used in this study (*Esr1^tm1Ks^* ^k^) completely eliminates the full length ERα protein, a small amount of a truncated ERα protein generated *via* alternative splicing is expressed from the *Esr1^tm1Ks^* ^k^ allele in at least some tissues (35). Although we cannot formally exclude the possibility that signaling through this truncated ERα protein may contribute to the attenuated loss of tolerance and immune cell activation observed in B6.*Sle1b.ERα^-/-^* mice, our previous studies using B6.*Sle1* congenic mice are not consistent with this idea. Here again, the observation that removal of either full length ERα or the ovaries, the primary source of estrogens, results in elimination of the female sex bias in loss of tolerance in B6.*Sle1* mice is most consistent with the idea that the loss of the full length ERα rather than expression of low levels of a truncated ERα in the *Esr1^tm1Ks^* ^k^ allele causes the attenuated loss of tolerance in B6.*Sle1.ERα^-/-^* females. Given that the female sex bias in B6.*Sle1* congenic mice is due almost exclusively to the action of *Sle1b* and the fact that all female sex biased effects of *Sle1b* are attenuated or eliminated as a result of the *Esr1^tm1Ks^* ^k^ allele, we conclude, by extension, that it is likely loss of full length ERα rather than the action of a truncated ERα protein that is responsible for loss the female sex biased phenotypes in B6.*Sle1b.ERα^-/-^* females. Further evidence arguing against the possibility that the truncated form of ERα contributes to the attenuation of autoimmunity associated with the *Esr1^tm1Ks^* ^k^ allele comes from previous studies demonstrating that both the *Esr1^tm1Ks^* ^k^ allele as well as a second allele that represents a complete knockout of ERα (*Esr1^tm4.2Ks^* ^k^) attenuate the development of lupus nephritis in females from the lupus-prone NZM2410 strain (44, 45).

In the study in NZM2410 mice involving the complete knockout of ERα, the ability of the *Esr1^tm4.2Ks^* ^k^ allele to reduce lupus nephritis was abrogated by ovariectomy and could not be restored by estrogen treatment, leading the authors to conclude that it was disruption of another component of the hypothalamic-pituitary-gonadal axis, possibly testosterone, rather than loss of ERα that reduced nephritis in NZM2410 mice carrying the complete knockout allele of ERα (45). By contrast, as discussed previously, our prior studies indicate that it is disruption of full length ERα and not a change in testosterone levels that results in attenuation of the autoimmune phenotype in B6.*Sle1.ERα^-/-^* and (NZB x NZW)F1 ERα^-/-^ mice (11, 13, 24). A plausible explanation for these differing observations and conclusions regarding the impact of disruption of ERα relates to differences in key aspects of the genetic background of the autoimmune prone strains used in these various studies. Of particular importance is likely to be the fact that in contrast to the (NZB x NZW)F1, B6.*Sle1*, and B6.*Sle1b* strains, each of which shows an autoimmune phenotype with a strong female sex bias, the NZM2410 strain does not show a female sex bias (46, 47). The lack of a female sex bias in the NZM2410 strain complicates the interpretation of the results of studies examining the impact of disruption of ERα and sex hormone manipulation in this strain. It is also important to note that although the *Esr1^tm1Ks^* ^k^ allele (knockout of full length ERα) and the *Esr1^tm4.2Ks^* ^k^ allele (complete knockout of ERα) both attenuated lupus nephritis in the NZM2410 strain, neither *ERα* knockout allele reduced anti-dsDNA autoantibodies in these mice (44, 45). These data indicate that in the NZM2410 strain, the protection against lupus nephritis conferred by these two *ERα* knockout alleles does not reflect effects of ERα on autoantibody production. Furthermore, Svenson et al. also showed that the *Esr1^tm1Ks^* ^k^ allele attenuated lupus nephritis in the lupus prone MRL-lpr strain without altering anti-dsDNA autoantibodies (44). These data in the NZM2410 and MRL-lpr strains are in sharp contrast to what we observed in the (NZB × NZW)F1 hybrid model, in which the attenuation of lupus nephritis due to the *Esr1^tm1Ks^* ^k^ allele (knockout of full length ERα) in female mice was associated with a significant decrease in anti-dsDNA IgG and anti-chromatin IgG autoantibodies (12). Likewise, in the B6.*Sle1* congenic strain, we again observed that the *Esr1^tm1Ks^* ^k^ allele (knockout of full length ERα) reduced anti-dsDNA IgG and anti-chromatin autoantibody development in female mice (24). Thus, in three strains showing a dramatic female sex bias in the loss of tolerance and the development of autoantibodies—the (NZB × NZW)F1 hybrid model, the B6.*Sle1* congenic strain, and the B6.*Sle1b* subcongenic strain—we find that the *Esr1^tm1Ks^* ^k^ allele (knockout of full length ERα) strongly reduces autoantibody development. These observations, taken together with those of Svenson et al. and Scott et al. in the NZM2410 and MRL-lpr strains, suggest that in genetic backgrounds that show a strong female sex bias, such as in (NZB × NZW)F1, B6.*Sle1*, and B6.*Sle1b* mice, disruption of ERα may reduce autoimmunity and/or lupus pathogenesis *via* mechanisms that are distinct from those in genetic backgrounds that do not show a female sex bias, such as the NZM2410 and MRL-lpr strains. In this context, it is also worth noting that both female and male NZM2410 and MRL-lpr mice develop lupus at younger ages than is seen in (NZB × NZW)F1 females (11, 46, 47). Thus, the extremely strong genetic predisposition for development of highly aggressive lupus nephritis in the NZM2410 and MRL-lpr strains may override the effects of endogenous sex hormones on lupus in these models.

In the present study, we focused on examining the role of ERα in mediating the female sex bias in the actions of the lupus susceptibility loci *Sle1a* and *Sle1b*. Although *Sle1a*-induced T cell hyperactivation shows a female sex bias, this phenotype is not modulated by disruption of ERα indicating that the female sex bias is not dependent upon ERα signaling. By contrast, disruption of ERα completely eliminated the female sex bias seen in *Sle1b*-induced T cell hyperactivation, expansion of the T_fh_ cell and germinal center B cell subsets, spontaneous germinal center formation, and development of anti-chromatin IgG autoantibodies. *Sle1b* also induces B cell hyperactivation in a female specific fashion, and this female specific B cell activation is abrogated by disruption of ERα. These data demonstrate that ERα signaling is responsible for the strong female sex bias in the phenotypes associated with the *Sle1b* lupus susceptibility locus. Our data also demonstrate that in the absence of ERα signaling, *Sle1b* is insufficient to induce B cell hyperactivation, suggesting that this phenotype requires the synergistic actions of ERα and *Sle1b*. Future studies aimed at understanding the basis for this synergy will help to uncover the molecular basis for the ability of ERα signaling to promote loss of tolerance, immune cell activation, and the development of autoimmunity and shed light on the causes of the strong female sex bias associated with these processes.

## Data Availability Statement

The raw data supporting the conclusions of this article will be made available by the authors, without undue reservation.

## Ethics Statement

The animal study was reviewed and approved by University of Nebraska Medical Center Institutional Animal Care and Use Committee.

## Author Contributions

KG conceived and designed the study. JG, SY, and KG performed the experiments, analyzed the data, and contributed to the interpretation of the data. JG and KG wrote the manuscript, and SY participated in the process. All authors contributed to the article and approved the submitted version.

## Funding

This work was supported by grants R01AI075167 and R01AI075167-S1 from the National Institute of Allergy and Infectious Diseases of the National Institutes of Health. This work was also supported by a University of Nebraska Medical Center institutional support grant.

## Conflict of Interest

The authors declare that the research was conducted in the absence of any commercial or financial relationships that could be construed as a potential conflict of interest.
