# Supplementary material for: Estrogen Receptor Alpha Signaling Is Responsible for the Female Sex Bias in the Loss of Tolerance and Immune Cell Activation Induced by the Lupus Susceptibility Locus Sle1b
Source: Front Immunol. 2020 Nov 10;11:582214. doi: 10.3389/fimmu.2020.582214 (PMC7683613; doi:10.3389/fimmu.2020.582214)
Supplement: Supplementary file 1 [file DataSheet_1.pdf]

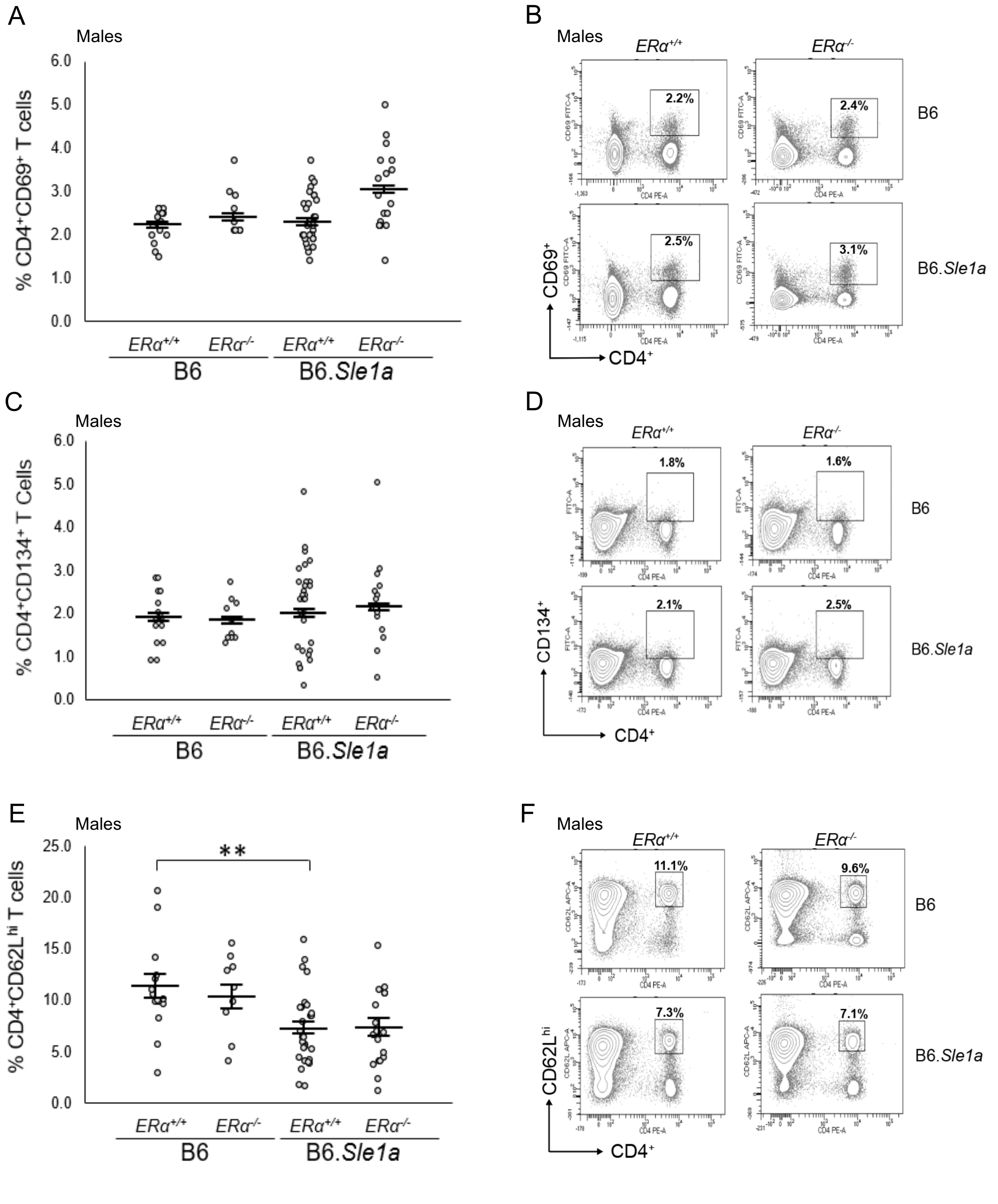

Supplementary Figure 1: Disruption of ERα does not impact T cell activation in B6.*Sle1a* males. (A) Dot plots show the percentage of splenocytes in male B6.*ERα*$^{+/+}$, B6.*ERα*$^{-/-}$, B6.*Sle1a*.*ERα*$^{+/+}$, and B6.*Sle1a*.*ERα*$^{-/-}$ mice that were CD4^{+}CD69^{+}. (B) Representative contour plots from show the frequency of CD4^{+}CD69^{+} T cells in male B6.*ERα*$^{+/+}$, B6.*ERα*$^{-/-}$,

B6.*Sle1a*.*ERα*$^{+/+}$, and B6.*Sle1a*.*ERα*$^{-/-}$ mice. (C) Dot plots show the percentage of splenocytes in male B6.*ERα*$^{+/+}$, B6.*ERα*$^{-/-}$, B6.*Sle1a*.*ERα*$^{+/+}$, and B6.*Sle1a*.*ERα*$^{-/-}$ mice that were $CD4^{+}C62L^{+}$ naïve T cells. (D) Representative contour plots show the frequency of $CD4^{+}CD62L^{+}$ T cells in male B6.*ERα*$^{+/+}$, B6.*ERα*$^{-/-}$, B6.*Sle1a*.*ERα*$^{+/+}$, and B6.*Sle1a*.*ERα*$^{-/-}$ mice. (E) Dot plots show the percentage of splenocytes in male B6.*ERα*$^{+/+}$, B6.*ERα*$^{-/-}$, B6.*Sle1a*.*ERα*$^{+/+}$, and B6.*Sle1a*.*ERα*$^{-/-}$ mice that were $CD4^{+}CD134^{+}$ activated T cells. (F) Representative contour plots show the frequency of $CD4^{+}CD134^{+}$ T cells in male B6.*ERα*$^{+/+}$, B6.*ERα*$^{-/-}$, B6.*Sle1a*.*ERα*$^{+/+}$, and B6.*Sle1a*.*ERα*$^{-/-}$ mice. Splenocytes were collected from male B6.*ERα*$^{+/+}$ (N=15), B6.*ERα*$^{-/-}$ (N=10), B6.*Sle1a*.*ERα*$^{+/+}$(N=30), and B6.*Sle1a*.*ERα*$^{-/-}$ (N=18) mice that were 5-6 months of age. In (A), (C), and (E), the longer horizontal bar in each panel denotes the mean for each group, and the shorter black bars indicate the standard error of the mean. The * indicates $p \leq 0.05$, and the ** indicates $p \leq 0.01$.
