# Supplementary material for: Estrogen Receptor Alpha Signaling Is Responsible for the Female Sex Bias in the Loss of Tolerance and Immune Cell Activation Induced by the Lupus Susceptibility Locus Sle1b
Source: Front Immunol. 2020 Nov 10;11:582214. doi: 10.3389/fimmu.2020.582214 (PMC7683613; doi:10.3389/fimmu.2020.582214)
Supplement: Supplementary file 2 [file DataSheet_2.pdf]

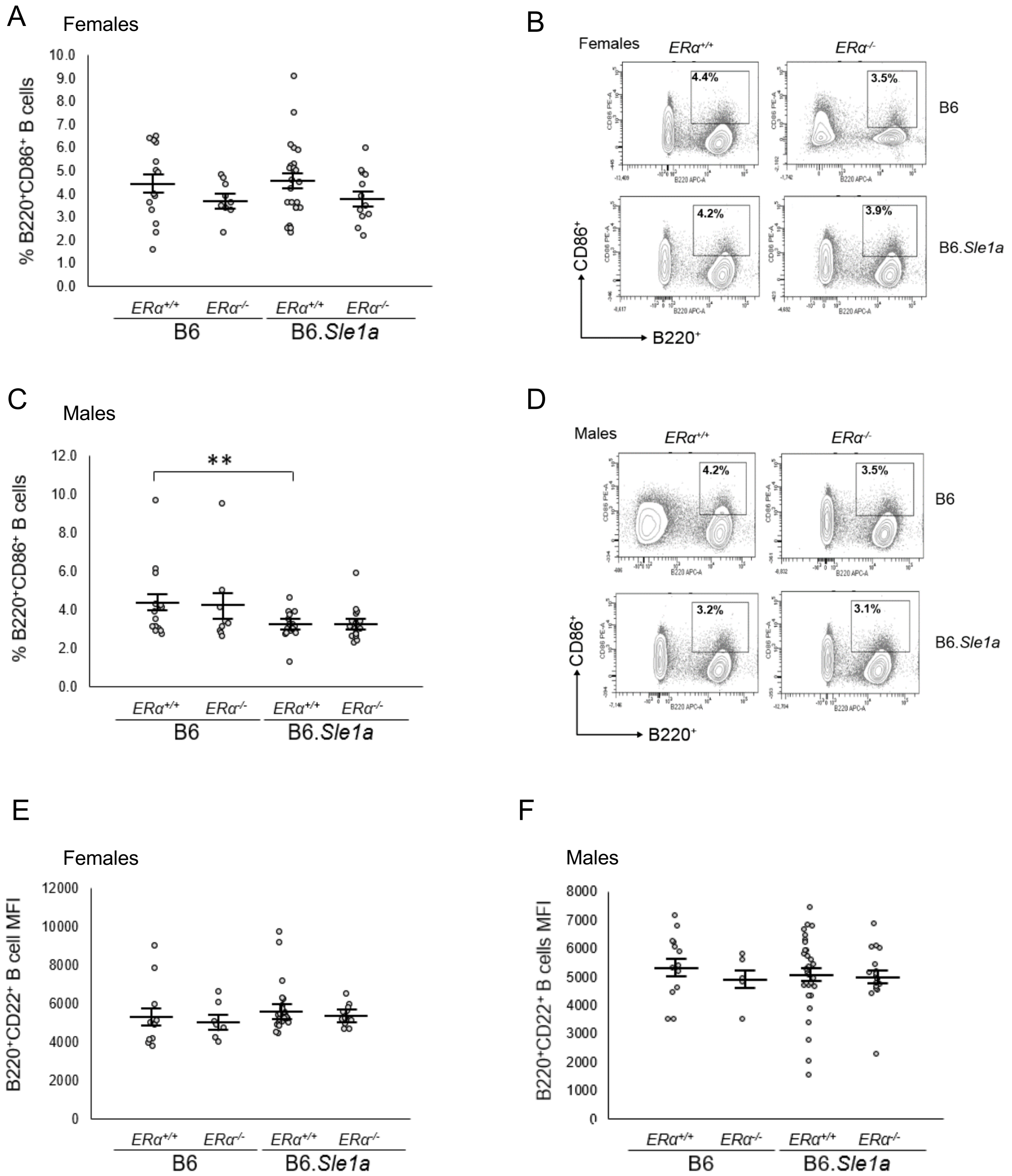

Supplementary Figure 2: *Sle1a* does not impact B cell activation and ERα does not modulate B cell activation in B6.*Sle1a* mice. (A) Dot plots show the percentage of splenocytes in female B6.$ER\alpha^{+/+}$ (N=14), B6.$ER\alpha^{-/-}$ (N=9), B6.*Sle1a*.$ER\alpha^{+/+}$ (N=24), and B6.*Sle1a*.$ER\alpha^{-/-}$

(N=12) mice that were B220$^+$CD86$^+$ activated B cells. (B) Representative contour plots from female B6.*ERα*$^{+/+}$, B6.*ERα*$^{-/-}$, B6.*Sle1a.ERα*$^{+/+}$, and B6.*Sle1a.ERα*$^{-/-}$ mice show the frequency of B220$^+$CD86$^+$ B cells. (C) Dot plots show the percentage of splenocytes in male mice B6.*ERα*$^{+/+}$ (N=15), B6.*ERα*$^{-/-}$ (N=9), B6.*Sle1a.ERα*$^{+/+}$ (N=16), and B6.*Sle1a.ERα*$^{-/-}$ (N=18) mice that were B220$^+$CD86$^+$ activated B cells. (D) Representative contour plots from male B6.*ERα*$^{+/+}$, B6.*ERα*$^{-/-}$, B6.*Sle1a.ERα*$^{+/+}$, and B6.*Sle1a.ERα*$^{-/-}$ mice show the frequency of B220$^+$CD86$^+$ B cells. (E) Dot plots show CD22 surface expression measured as mean fluorescence intensity (MFI) in B220$^+$CD22$^+$ B cells in female B6.*ERα*$^{+/+}$ (N=10), B6.*ERα*$^{-/-}$ (N=7), B6.*Sle1a.ERα*$^{+/+}$ (N=24), and B6.*Sle1a.ERα*$^{-/-}$ (N=13) mice. (F) Dot plots show CD22 surface expression measured as mean fluorescence intensity (MFI) in B220$^+$CD22$^+$ B cells in male B6.*ERα*$^{+/+}$ (N=13), B6.*ERα*$^{-/-}$ (N=5), B6.*Sle1a.ERα*$^{+/+}$ (N=30), and B6.*Sle1a.ERα*$^{-/-}$ (N=18) mice is shown. Splenocytes were collected from mice that were 5-6 months of age. The longer horizontal bar in each panel denotes the mean for each group, and the shorter black bars indicate the standard error of the mean. The * indicates $p \leq 0.05$, and the ** indicates $p \leq 0.01$.
