# Supplementary material for: Estrogen Receptor Alpha Signaling Is Responsible for the Female Sex Bias in the Loss of Tolerance and Immune Cell Activation Induced by the Lupus Susceptibility Locus Sle1b
Source: Front Immunol. 2020 Nov 10;11:582214. doi: 10.3389/fimmu.2020.582214 (PMC7683613; doi:10.3389/fimmu.2020.582214)
Supplement: Supplementary file 3 [file DataSheet_3.pdf]

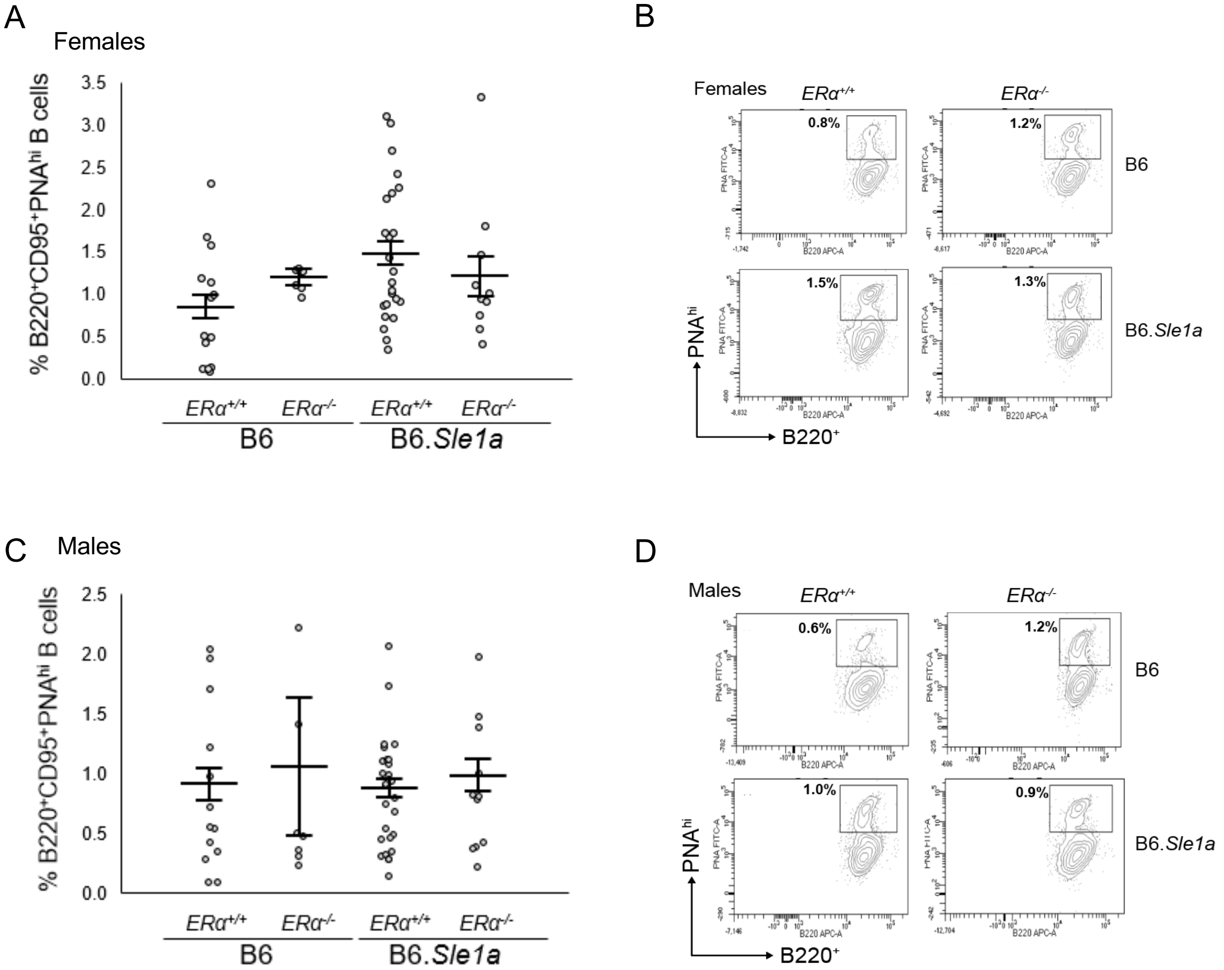

Supplementary Figure 3: *Sle1a* has no significant impact on the size of the germinal center B cell population. (A) Dot plots show the percentage of splenocytes in female B6.$ER\alpha^{+/+}$ (N=14), B6.$ER\alpha^{-/-}$ (N=7), B6.*Sle1a*.$ER\alpha^{+/+}$ (N=24), and B6.*Sle1a*.$ER\alpha^{-/-}$ (N=10) mice that were $B220^{+}CD95^{+}PNA^{hi}$ germinal center B cells. (B) Representative contour plots from female B6.$ER\alpha^{+/+}$, B6.$ER\alpha^{-/-}$, B6.*Sle1a*.$ER\alpha^{+/+}$, and B6.*Sle1a*.$ER\alpha^{-/-}$ mice show the frequency of $B220^{+}CD95^{+}PNA^{hi}$ germinal center B cells. (C) Dot plots show the percentage of splenocytes in male B6.$ER\alpha^{+/+}$ (N=13), B6.$ER\alpha^{-/-}$ (N=7), B6.*Sle1a*.$ER\alpha^{+/+}$ (N=30), and B6.*Sle1a*.$ER\alpha^{-/-}$ (N=11) mice that were $B220^{+}CD95^{+}PNA^{hi}$ germinal center B cells. (D) Representative contour plots from male B6.$ER\alpha^{+/+}$, B6.$ER\alpha^{-/-}$, B6.*Sle1a*.$ER\alpha^{+/+}$, and B6.*Sle1a*.$ER\alpha^{-/-}$ mice show the frequency of $B220^{+}CD95^{+}PNA^{hi}$ germinal center B cells. Splenocytes were collected from mice that were 5-6 months of age. The longer horizontal bar in each panel denotes the mean for each group, and the shorter black bars indicate the standard error of the mean. The * indicates $p \leq 0.05$, and the ** indicates $p \leq 0.01$.
