# Supplementary material for: Estrogen Receptor Alpha Signaling Is Responsible for the Female Sex Bias in the Loss of Tolerance and Immune Cell Activation Induced by the Lupus Susceptibility Locus Sle1b
Source: Front Immunol. 2020 Nov 10;11:582214. doi: 10.3389/fimmu.2020.582214 (PMC7683613; doi:10.3389/fimmu.2020.582214)
Supplement: Supplementary file 4 [file DataSheet_4.pdf]

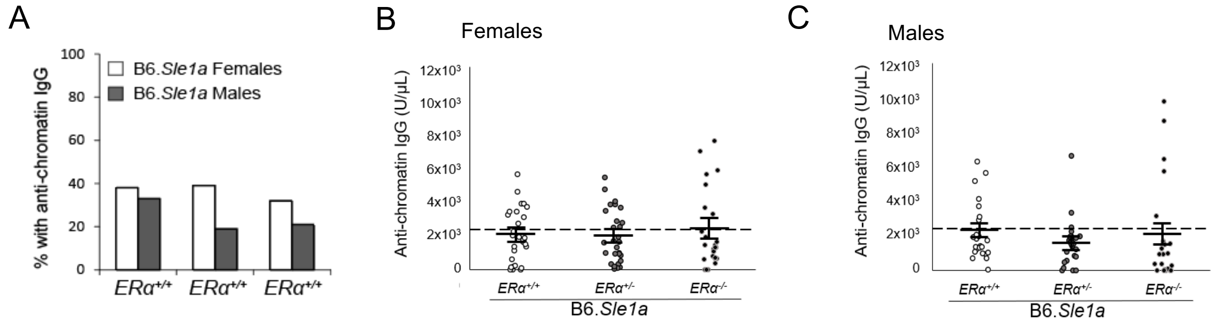

Supplementary Figure 4: Disruption of ERα has no impact on loss of tolerance in B6.*Sle1a* congenic mice (A) The proportion of B6.*Sle1a* female and male mice of all three ERα genotypes ($ER\alpha^{+/+}$, $ER\alpha^{+/-}$, $ER\alpha^{-/-}$) producing anti-chromatin IgG autoantibodies at 5 months of age is shown. (B) The concentration of anti-chromatin IgG autoantibodies in female $ER\alpha^{+/+}$ (N=29), $ER\alpha^{+/-}$ (N=28), and $ER\alpha^{-/-}$ (N=25) B6.*Sle1a* congenic mice is shown. (C) The concentration of anti-chromatin IgG autoantibodies in male $ER\alpha^{+/+}$ (N=24), $ER\alpha^{+/-}$ (N=27), and $ER\alpha^{-/-}$ (N=24) B6.*Sle1a* congenic mice is shown. In (B) and (C), the dashed line represents the threshold used to designate a positive autoantibody titer in the experimental mice. This threshold was set at 2 standard deviations above the mean of a group of age-matched control B6 mice as has been described previously (23, 24). The longer black horizontal bar indicates the mean for each group, and the shorter black bars indicate the standard error of the mean. The * indicates p≤0.05, and the ** indicates p≤0.01.
