# Supplementary material for: Estrogen Receptor Alpha Signaling Is Responsible for the Female Sex Bias in the Loss of Tolerance and Immune Cell Activation Induced by the Lupus Susceptibility Locus Sle1b
Source: Front Immunol. 2020 Nov 10;11:582214. doi: 10.3389/fimmu.2020.582214 (PMC7683613; doi:10.3389/fimmu.2020.582214)
Supplement: Supplementary file 5 [file DataSheet_5.pdf]

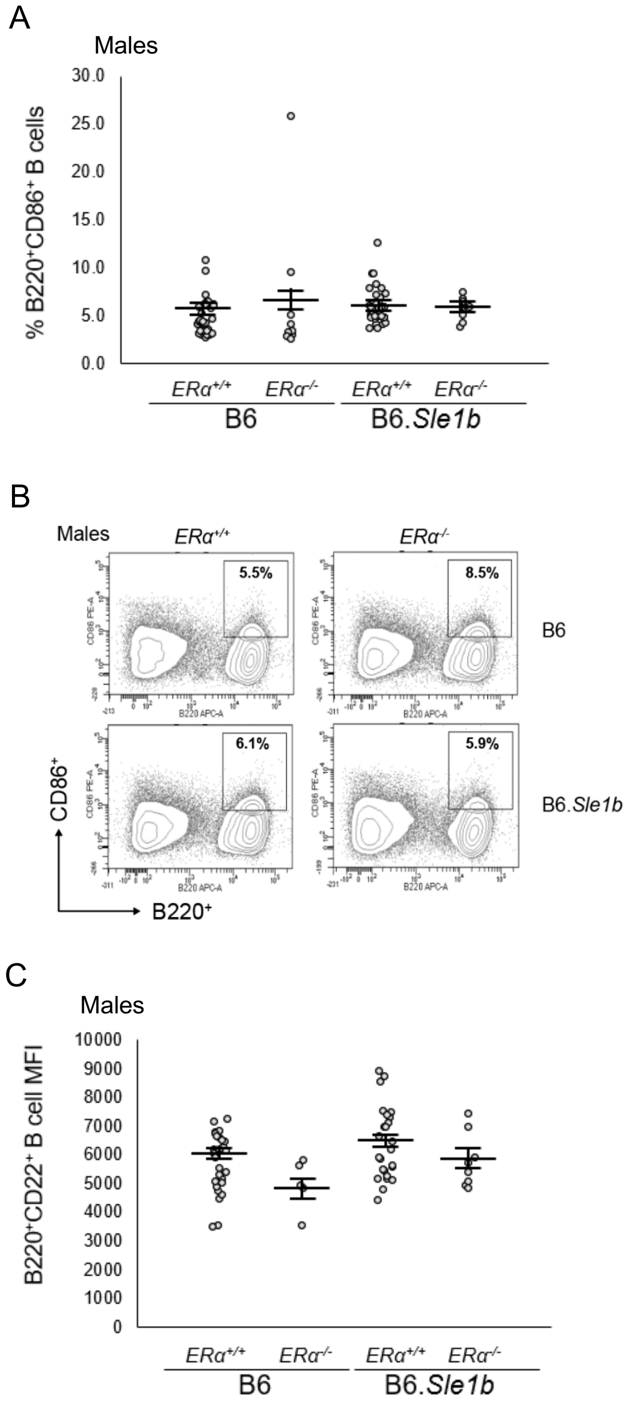

Supplementary Figure 5: Neither *Sle1b* nor ERα impacts B cell activation in males
(A) Dot plots show the percentage of splenocytes in male B6.*ERα*$^{+/+}$ (N=32), B6.*ERα*$^{-/-}$ (N=10), B6.*Sle1b*.*ERα*$^{+/+}$ (N=32), and B6.*Sle1b*.*ERα*$^{-/-}$ (N=11)mice that were B220^{+}CD86^{+} activated B cells. (B) Representative contour plots from show the frequency of B220^{+}CD86^{+} B cells in male B6.*ERα*$^{+/+}$, B6.*ERα*$^{-/-}$, B6.*Sle1b*.*ERα*$^{+/+}$, and B6.*Sle1b*.*ERα*$^{-/-}$ mice. (C) Dot plots show CD22

surface expression measured as mean fluorescence intensity (MFI) in B220$^+$CD22$^+$ B cells in male B6.*ERα*$^{+/+}$ (N=27), B6.*ERα*$^{-/-}$, (N=5) B6.*Sle1b*.*ERα*$^{+/+}$ (N=25), and B6.*Sle1b*.*ERα*$^{-/-}$ (N=8) mice is shown. Splenocytes were collected from mice that were 5-6 months of age. The longer horizontal bar in each panel denotes the mean for each group, and the shorter black bars indicate the standard error of the mean. The * indicates p≤0.05, and the ** indicates p≤0.01.
