# Supplementary material for: Estrogen Receptor Alpha Signaling Is Responsible for the Female Sex Bias in the Loss of Tolerance and Immune Cell Activation Induced by the Lupus Susceptibility Locus Sle1b
Source: Front Immunol. 2020 Nov 10;11:582214. doi: 10.3389/fimmu.2020.582214 (PMC7683613; doi:10.3389/fimmu.2020.582214)
Supplement: Supplementary file 7 [file DataSheet_7.pdf]

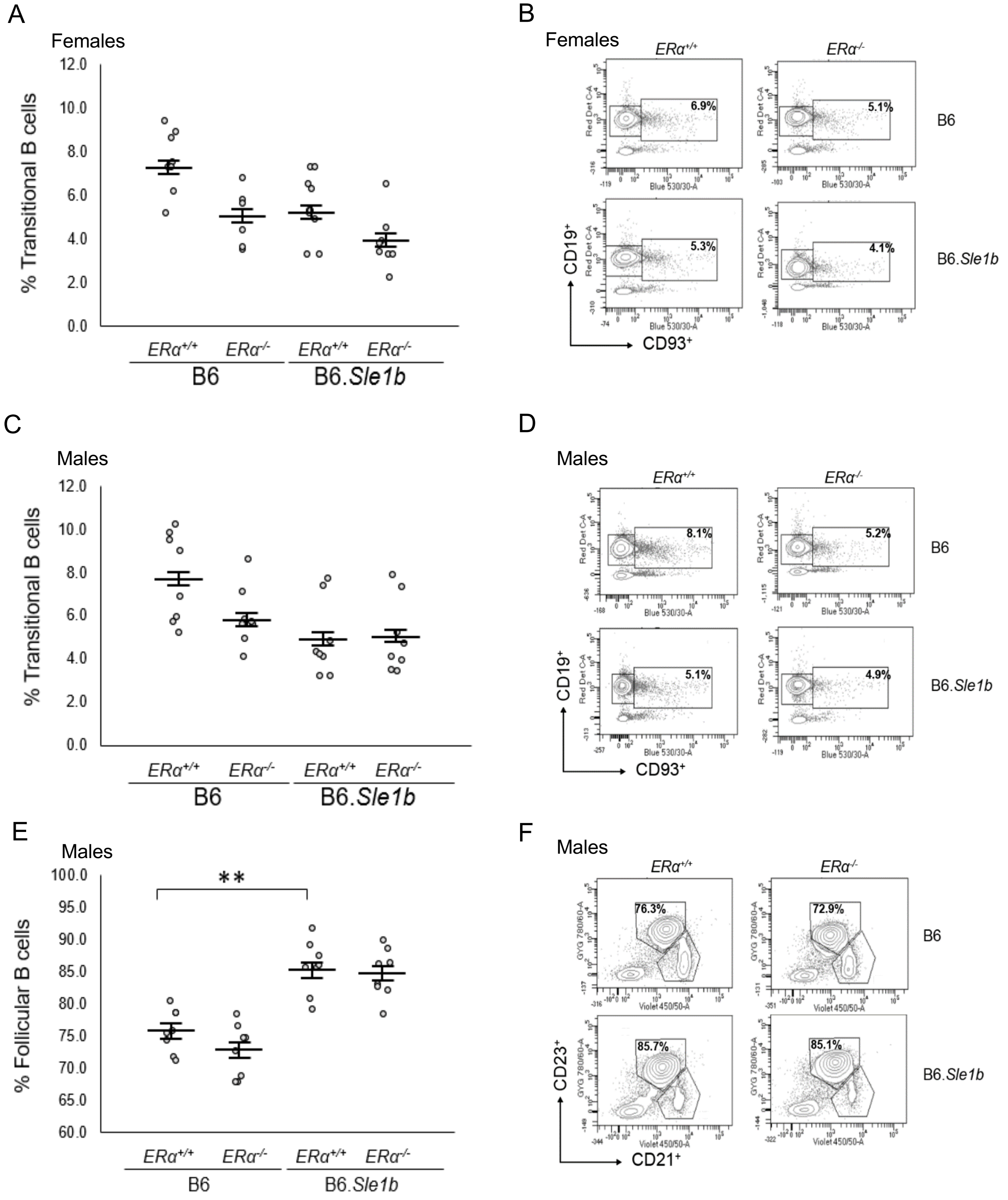

Supplementary Figure 7: ERα deficiency has no significant impact on transitional B cell development in B6.*Sle1b* congenic mice or follicular B cell development in B6.*Sle1b* congenic males. (A) Dot plots show the percentage of splenic transitional B cells (identified as

lymphocyte singlets that were CD5$^-$CD19$^+$CD93$^+$) in female B6.*ERα*$^{+/+}$, B6.*ERα*$^{-/-}$, B6.*Sle1b.ERα*$^{+/+}$, and B6.*Sle1b.ERα*$^{-/-}$ mice. (B) Representative contour plots from show the frequency of transitional B cells in female B6.*ERα*$^{+/+}$, B6.*ERα*$^{-/-}$, B6.*Sle1b.ERα*$^{+/+}$, and B6.*Sle1b.ERα*$^{-/-}$ mice. (C) Dot plots show the percentage of splenocytes in male B6.*ERα*$^{+/+}$, B6.*ERα*$^{-/-}$, B6.*Sle1b.ERα*$^{+/+}$, and B6.*Sle1b.ERα*$^{-/-}$ mice that were transitional B cells. (D) Representative contour plots from show the frequency of transitional B cells in male B6.*ERα*$^{+/+}$, B6.*ERα*$^{-/-}$, B6.*Sle1b.ERα*$^{+/+}$, and B6.*Sle1b.ERα*$^{-/-}$ mice. E) Dot plots show the percentage of splenic follicular B cells (identified as lymphocyte singlets that were CD5$^-$CD19$^+$CD93$^-$CD21$^-$CD23$^+$) in male B6.*ERα*$^{+/+}$, B6.*ERα*$^{-/-}$, B6.*Sle1b.ERα*$^{+/+}$, and B6.*Sle1b.ERα*$^{-/-}$ mice. (F) Representative contour plots from show the frequency of follicular B cells in male B6.*ERα*$^{+/+}$, B6.*ERα*$^{-/-}$, B6.*Sle1b.ERα*$^{+/+}$, and B6.*Sle1b.ERα*$^{-/-}$ mice. Splenocytes were collected from mice that were 5-6 months of age. The longer horizontal bar in each panel denotes the mean for each group (N=8 per group), and the shorter black bars indicate the standard error of the mean. The ** indicates $p \leq 0.01$.
