# Supplementary material for: Estrogen Receptor Alpha Signaling Is Responsible for the Female Sex Bias in the Loss of Tolerance and Immune Cell Activation Induced by the Lupus Susceptibility Locus Sle1b
Source: Front Immunol. 2020 Nov 10;11:582214. doi: 10.3389/fimmu.2020.582214 (PMC7683613; doi:10.3389/fimmu.2020.582214)
Supplement: Supplementary file 8 [file DataSheet_8.pdf]

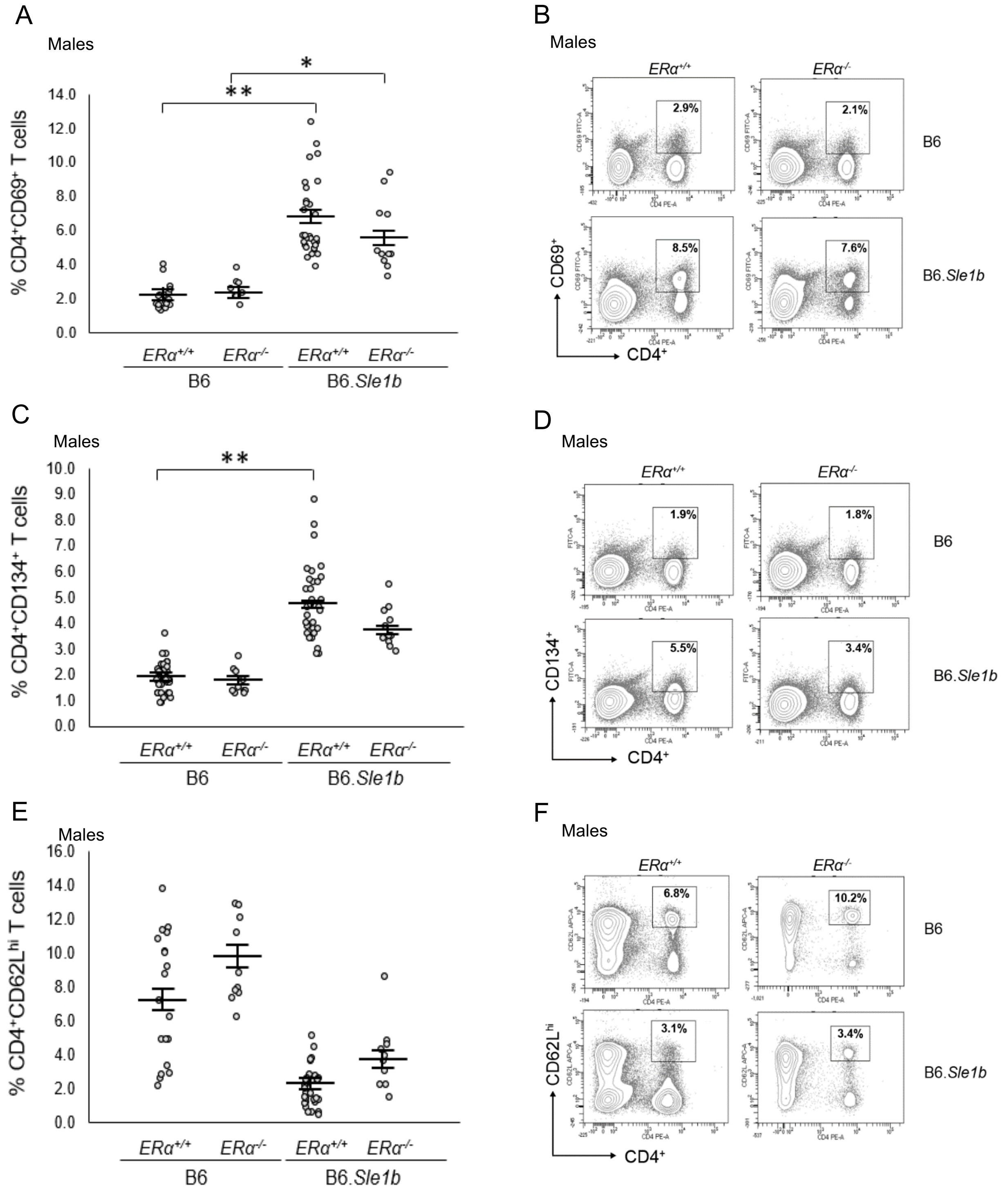

Supplementary Figure 8: Disruption of ERα does not impacts T cell hyperactivation in B6.*Sle1b* males (A) Dot plots show the percentage of splenocytes in male B6.*ERα*$^{+/+}$, B6.*ERα*$^{-/-}$, B6.*Sle1b*.*ERα*$^{+/+}$, and B6.*Sle1b*.*ERα*$^{-/-}$ mice that were CD4^{+}CD69^{+} activated T cells. (B)

Representative contour plots from show the frequency of CD4^{+}CD69^{+} T cells in male B6.*ERα*$^{+/+}$, B6.*ERα*$^{-/-}$, B6.*Sle1b.ERα*$^{+/+}$, and B6.*Sle1b.ERα*$^{-/-}$ mice. (C) Dot plots show the percentage of splenocytes in male B6.*ERα*$^{+/+}$, B6.*ERα*$^{-/-}$, B6.*Sle1b.ERα*$^{+/+}$, and B6.*Sle1b.ERα*$^{-/-}$ mice that were CD4^{+}CD134^{+} activated T cells. (D) Representative contour plots from show the frequency of CD4^{+}CD134^{+} T cells in male B6.*ERα*$^{+/+}$, B6.*ERα*$^{-/-}$, B6.*Sle1b.ERα*$^{+/+}$, and B6.*Sle1b.ERα*$^{-/-}$ mice. (E) Dot plots show the percentage of splenocytes in male B6.*ERα*$^{+/+}$, B6.*ERα*$^{-/-}$, B6.*Sle1b.ERα*$^{+/+}$, and B6.*Sle1b.ERα*$^{-/-}$ mice that were CD4^{+}CD62L^{+} naïve T cells. (F) Representative contour plots from show the frequency of CD4^{+}CD62L^{+} T cells in male B6.*ERα*$^{+/+}$, B6.*ERα*$^{-/-}$, B6.*Sle1b.ERα*$^{+/+}$, and B6.*Sle1b.ERα*$^{-/-}$ mice. Splenocytes were collected from male B6.*ERα*$^{+/+}$ (N=32), B6.*ERα*$^{-/-}$ (N=10), B6.*Sle1b.ERα*$^{+/+}$ (N=32), and B6.*Sle1b.ERα*$^{-/-}$ (N=11) mice that were 5-6 months of age. The longer horizontal bar in each panel denotes the mean for each group, and the shorter black bars indicate the standard error of the mean. The * indicates $p \leq 0.05$, and the ** indicates $p \leq 0.01$.
